# Cophenetic metrics for phylogenetic trees, after Sokal and Rohlf

**DOI:** 10.1186/1471-2105-14-3

**Published:** 2013-01-16

**Authors:** Gabriel Cardona, Arnau Mir, Francesc Rosselló, Lucía Rotger, David Sánchez

**Affiliations:** 1 Department of Mathematics and Computer Science, University of the Balearic Islands, E-07122 Palma de Mallorca, Spain

## Abstract

**Background:**

Phylogenetic tree comparison metrics are an important tool in the study of evolution, and hence the definition of such metrics is an interesting problem in phylogenetics. In a paper in Taxon fifty years ago, Sokal and Rohlf proposed to measure quantitatively the difference between a pair of phylogenetic trees by first encoding them by means of their half-matrices of cophenetic values, and then comparing these matrices. This idea has been used several times since then to define dissimilarity measures between phylogenetic trees but, to our knowledge, no proper metric on weighted phylogenetic trees with nested taxa based on this idea has been formally defined and studied yet. Actually, the cophenetic values of pairs of different taxa alone are not enough to single out phylogenetic trees with weighted arcs or nested taxa.

**Results:**

For every (rooted) phylogenetic tree *T*, let its *cophenetic vectorφ*(*T*) consist of all pairs of cophenetic values between pairs of taxa in *T* and all depths of taxa in *T*. It turns out that these cophenetic vectors single out weighted phylogenetic trees with nested taxa. We then define a family of cophenetic metrics *d*_*φ*,*p*_ by comparing these cophenetic vectors by means of *L*^*p*^ norms, and we study, either analytically or numerically, some of their basic properties: neighbors, diameter, distribution, and their rank correlation with each other and with other metrics.

**Conclusions:**

The cophenetic metrics can be safely used on weighted phylogenetic trees with nested taxa and no restriction on degrees, and they can be computed in *O*(*n*^2^) time, where *n* stands for the number of taxa. The metrics *d*_*φ*,1_ and *d*_*φ*,2_ have positive skewed distributions, and they show a low rank correlation with the Robinson-Foulds metric and the nodal metrics, and a very high correlation with each other and with the splitted nodal metrics. The diameter of *d*_*φ*,*p*_, for p⩾1 , is in *O*(*n*^(*p*+2)/*p*^), and thus for low *p* they are more discriminative, having a wider range of values.

## Background

Many phylogenetic trees published in the literature or included in phylogenetic databases are actually alternative phylogenies for the same sets of organisms, obtained from different datasets or using different evolutionary models or different phylogenetic reconstruction algorithms [1]. This variety of phylogenetic trees makes it necessary to develop methods for measuring their differences [2, Chapter 30]. The comparison of phylogenetic trees is also used to compare phylogenetic trees obtained through numerical algorithms with other types of hierarchical classifications [3,4], to assess the stability of reconstruction methods [5], and in the comparative analysis of dendrograms and other hierarchical cluster structures [6,7]. Hence, and since the safest way to quantify the differences between a pair of trees is through a metric, “tree comparison metrics are an important tool in the study of evolution” [8].

Many metrics for the comparison of phylogenetic trees have been proposed so far [2, Chapter 30]. Some of these metrics are edit distances that count how many operations of a given type are necessary to transform one tree into the other. These metrics include the nearest-neighbor interchange metric [9] and the subtree prune-and-regrafting distance [10]. Other metrics compare a pair of phylogenetic trees through some consensus subtree. This is the case for instance of the MAST distances defined in [11-13]. Finally, many metrics for phylogenetic trees are based on the comparison of encodings of the phylogenetic trees, like for instance the Robinson-Foulds metric [14,15] (which can also be understood as an edit distance), the triples metric [16], the classical nodal metrics for binary phylogenetic trees [5,8,17-19], and the splitted nodal metrics for arbitrary phylogenetic trees [20]. The advantage of this last kind of metrics is that, unlike the edit and the consensus distances, they are usually computed in low polynomial time.

In an already fifty years old paper [4], Sokal and Rohlf proposed a technique to compare dendrograms (which, in their paper, were equivalent to weighted phylogenetic trees without nested taxa) on the same set of taxa, by encoding them by means of their half-matrices of cophenetic values, and then comparing these structures. Their method runs as follows. To begin with, they divide the range of depths of internal nodes in the tree into a suitable number of equal intervals and number increasingly these intervals. Then, for each pair of taxa *i,j* in the tree, they compute their *cophenetic value* as the class mark of the interval where the depth of their lowest common ancestor lies. Then, to compare two phylogenetic trees, they compare their corresponding half-matrices of cophenetic values. In that paper, they do it specifically by calculating a correlation coefficient between their entries. Sokal and Rohlf’s paper [4] is quite cited (612 cites according to Google Scholar on July 1, 2012) and their method has been often used to compare hierarchical classifications (see, for instance, [21-23]).

Since Sokal and Rohlf’s paper, other papers have compared the half-matrices of cophenetic values to define dissimilarity measures between phylogenetic trees (see, for instance, [3,24]), and such half-matrices have also been used in the so-called “comparative method”, the statistical methods used to make inferences on the evolution of a trait among species from the distribution of other traits: see [25,26] and [2, Chapter 25]. But, to our knowledge, no proper metric for phylogenetic trees based on cophenetic values has been formally defined and studied in the literature. In this paper we define a new family of metrics for weighted phylogenetic trees with nested taxa based on Sokal and Rohlf’s idea and we study some of their basic properties: neighbors, diameter, distribution, and their rank correlation with each other and with other metrics.

Our approach differs in some minor points with Sokal and Rohlf’s. For instance, we use as the cophenetic value *φ*(*i*,*j*) of a pair of taxa *i,j* the actual depth of the lowest common ancestor of *i* and *j*, instead of class marks, which was done by Sokal and Rohlf because of practical limitations. Moreover, instead of using a correlation coefficient, we define metrics by using *L*^*p*^ norms. Finally, we do not restrict ourselves to dendrograms, without internal labeled nodes, but we also allow nested taxa.

**Figure 1:**
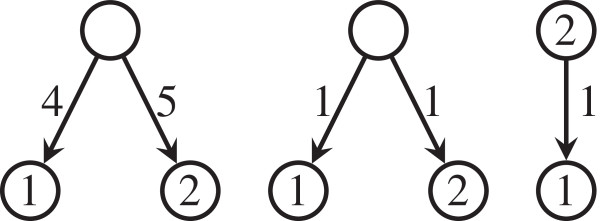
An unweighted phylogenetic tree on 7 taxa.

There is, however, a main difference between our approach and Sokal and Rohlf’s. We do not only consider the cophenetic values of pairs of taxa, but also the depths of the taxa. We must do so because we want to define a metric, where zero distance means isomorphism, and the cophenetic values of pairs of different taxa alone do not single out even the dendrograms considered by Sokal and Rohlf. That is, two non isomorphic weighted phylogenetic trees without nested taxa on the same set of taxa can have the same vectors of cophenetic values; see Figure 1.

It turns out that the *cophenetic vector* consisting of all cophenetic values of pairs of taxa and the depths of all taxa characterizes a weighted phylogenetic tree with nested taxa. This fact comes from the well known relationship between cophenetic values and patristic distances. If we denote by *δ*(*i*) the depth of a taxon *i*, by *φ*(*i*,*j*) the cophenetic value of a pair of taxa *i,j* and by *d*(*i*,*j*) the distance between *i* and *j*, then [27]

d(i,j)=δ(i)+δ(j)-2φ(i,j).

So, if the depths of the taxa are known, the knowledge of the cophenetic values of pairs of taxa is equivalent to the knowledge of the additive distance defined by the tree. On their turn, the depths and the additive distance single out the unrooted semi-labelled weighted tree associated to the phylogenetic tree with the former root labeled with a specific label “root”, and hence the phylogenetic tree itself: cf. Theorem 1.

The fact that cophenetic vectors single out weighted phylogenetic trees with nested taxa can also be deduced from their relationship with splitted path lengths [20]. Recall that the splitted path length *ℓ*(*i*, *j*) is the distance from the lowest common ancestor of *i* and *j* to *i*. It is known [20, Thm. 10] that the matrix (*ℓ*(*i*, *j*))_*i*,*j*_ characterizes a weighted phylogenetic tree with nested taxa. Since, obviously,

ℓ(i,j)=δ(i)-φ(i,j),

the cophenetic vector uniquely determines the matrix of splitted path lengths, and hence the tree.^a^

The vector of cophenetic values of pairs of different taxa is also related to the notion of ultrametric [28,29]. Indeed, notice that -*φ* satisfies the three-point condition of ultrametrics: for every taxa *i*, *j*, *k*,

-φ(i,j)⩽min{-φ(i,k),-φ(j,k)}.

But -*φ* is not an ultrametric, as *φ*(*i*, *i*) = *δ*(*i*) ≠ 0. Actually, *φ* can only be used to define an ultrametric precisely on ultrametric trees, where the depths of all leaves are the same, say *Δ*. In this case, *Δ* - *φ* is the ultrametric defined by the tree. In particular, ultrametric trees can be compared by comparing their vectors of cophenetic values of pairs of different taxa. A similar idea is used in [30] to induce an average genetic distance between populations from the average coancestry coefficient.

We would like to dedicate this paper to the memory of Robert R. Sokal, father of the field of numerical taxonomy and who passed away last April. His ideas permeate biostatistics and computational phylogenetics.

### Notations

A *rooted tree* is a directed finite graph that contains a distinguished node, called the *root*, from which every node can be reached through exactly one path. A *weighted rooted tree* is a pair (*T*, *ω*) consisting of a rooted tree *T* = (*V*, *E*) and a *weight function*$\omega :E\to R_{>0}$ that associates to every arc *e* ∈ *E* a non-negative real number *ω*(*e*) > 0. We identify every *unweighted* (that is, where no weight function has been explicitly defined) rooted tree *T* with the weighted rooted tree (*T*, *ω*) with *ω* the weight 1 constant function.

Let *T* = (*V*, *E*) be a rooted tree. Whenever (*u*, *v*) ∈ *E*, we say that *v* is a *child* of *u* and that *u* is the *parent* of *v*. Two nodes with the same parent are *siblings*. The nodes without children are the *leaves* of the tree, and the other nodes (including the root) are called *internal*. A *pendant arc* is an arc ending in a leaf. The nodes with exactly one child are called *elementary*. A tree is *binary*, or *fully resolved*, when every internal node has exactly two children.

Whenever there exists a path from a node *u* to a node *v*, we shall say that *v* is a *descendant* of *u* and also that *u* is an *ancestor* of *v*, and we shall denote it by *v* ≼ *u*; if, moreover, *u* ≠ *v*, we shall write *v* ≺ *u*. The *lowest common ancestor* (LCA) of a pair of nodes *u, v* of a rooted tree *T*, in symbols [*u*, *v*]_*T*_, is the unique common ancestor of them that is a descendant of every other common ancestor of them. Given a node *v* of a rooted tree *T*, the *subtree of T rooted at v* is the subgraph of *T* induced on the set of descendants of *v* (including *v* itself). A rooted subtree is a *cherry* when it has 2 leaves, a *triplet*, when it has 3 leaves, and a *quartet*, when it has 4 leaves.

The *distance* from a node *u* to a descendant *v* of it in a weighted rooted tree *T* is the sum of the weights of the arcs in the unique path from *u* to *v*. In an unweighted rooted tree, this distance is simply the number of arcs in this path. The *depth* of a node *v*, in symbols *δ*_*T*_(*v*), is the distance from the root to *v*.

Let *S* be a non-empty finite set of *labels*, or *taxa*. A (*weighted*) *phylogenetic tree* on *S* is a (weighted) rooted tree with some of its nodes bijectively labeled in the set *S*, including all its leaves and all its elementary nodes except possibly the root (which can be elementary but unlabeled). The reasons why we allow unlabeled elementary roots are that our results are still valid for phylogenetic trees containing them, and that even if we forbid them, we would need in some proofs to use that Theorem 1 below is true for phylogenetic trees containing them. Moreover, it is not uncommon to add an unlabeled elementary root to a phylogenetic tree in some contexts: see, for instance, the phylogenetic trees depicted in Wikipedia’s entry “Phylogenetic tree”.^b^

In a phylogenetic tree, we shall always identify a labeled node with its taxon. The internal labeled nodes of a phylogenetic tree are called *nested taxa*. Notice in particular that a phylogenetic tree without nested taxa cannot have elementary nodes other than the root. Although in practice *S* may be any set of taxa, to fix ideas we shall usually take *S* = {1, …, *n*}, with *n* the number of labeled nodes of the tree, and we shall use the term *phylogenetic tree with n taxa* to refer to a phylogenetic tree on this set.

Given a set *S* of taxa, we shall consider the following spaces of phylogenetic trees: 

• WT(S) , of all weighted phylogenetic trees on *S*

• UT(S) , of all unweighted phylogenetic trees on *S*

• T(S) , of all unweighted phylogenetic trees on *S* without nested taxa

• BT(S) , of all binary unweighted phylogenetic trees on *S* without nested taxa

When *S* = {1, …, *n*}, we shall simply write ${WT}_{n}$, ${UT}_{n}$, $T_{n}$, and ${BT}_{n}$, respectively.

Two phylogenetic trees *T* and *T*^′^ on the same set *S* of taxa are *isomorphic* when they are isomorphic as directed graphs and the isomorphism sends each labeled node of *T* to the labeled node with the same label in *T*^′^. An isomorphism of weighted phylogenetic trees is also required to preserve arc weights. We shall make the abuse of notation of saying that two isomorphic trees are actually the same, and hence of denoting that two trees *T*, *T*^′^ are isomorphic by simply writing *T* = *T*^′^.

## Methods

### Cophenetic vectors

Let *S* be henceforth a non-empty set of taxa with |*S*| = *n*, which without any loss of generality we identify with {1, …, *n*}. Let $T\in {WT}_{n}$ be a weighted phylogenetic tree on *S*. For every pair of different taxa *i, j* in *T*, their *cophenetic value* is the depth of their LCA:

$$\varphi_{T}(i,j)=\delta_{T}({\left[i,j\right]}_{T}).$$

To simplify the notations, we shall often write *φ*_*T*_(*i*, *i*) to denote the depth *δ*_*T*_(*i*) of a taxon *i*.

The *cophenetic vector* of *T* is

$$\varphi (T)={\left(\varphi_{T}(i,j)\right)}_{1⩽i⩽j⩽n}\in R^{n(n+1)/2},$$

with its elements lexicographically ordered in (*i*, *j*).

#### Example 1

**Figure 2:**
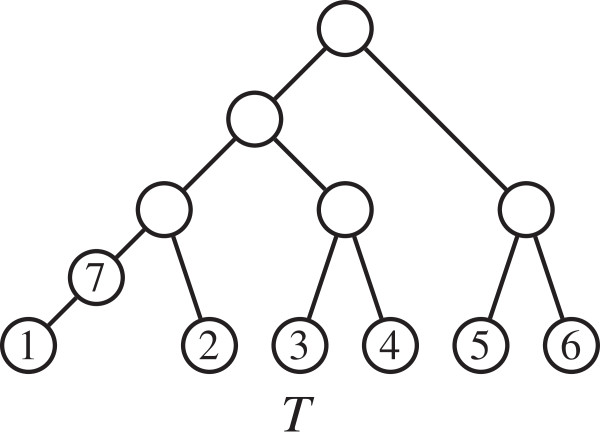
**Three non-isomorphic trees with the same vector**$\tilde{\varphi }(T)$**.**

If *T* is the unweighted phylogenetic tree in Figure 2, then *φ*(*T*) is the vector obtained by lexicographically ordering in (*i*, *j*) the elements of Table 1.

**Table 1:** **Cophenetic values of the pairs of taxa in the phylogenetic tree *T *in Figure**2

**j**	**1**	**2**	**3**	**4**	**5**	**6**	**7**
**i**							
1	4	2	1	1	0	0	3
2		3	1	1	0	0	2
3			3	2	0	0	1
4				3	0	0	1
5					2	1	0
6						2	0
7							3

The cophenetic vectors single out weighted phylogenetic trees with nested taxa.

#### Theorem 1

For every $T,T^{\prime }\in WT(S)$, if *φ*(*T*) = *φ*(*T*^′^), then *T* = *T*^′^.

#### Proof

Let *r* be a symbol not belonging to *S* and let *X* = *S* ∪ {*r*}. Recall that a *weighted X-tree* is an undirected weighted tree *T* with set of nodes *V* endowed with a (non necessarily injective) node-labeling mapping *f* : *X* → *V* such that *f*(*X*) contains all the leaves and all the degree-2 nodes in *T*[31].

For every T∈WT(S), let *T*^∗^ be the weighted *X*-tree obtained by considering *T* as undirected and adding to its former root the label *r*. Then, the distance $d_{T^{*}}$ on *T*^∗^ between pairs of labels in *X* is uniquely determined by *φ*(*T*) in the following way:

$$\;\begin{array}{l} d_{T^{*}}(i,r)=\delta_{T}(i)\;\text{for every}\;i\in S\\ d_{T^{*}}(i,j)=\delta_{T}(i)+\delta_{T}(j)-2\varphi_{T}(i,j)\;\text{for every}\;i,j\in S \end{array}$$

Now, *T*^∗^ is singled out by $d_{T^{*}}$[31, Thm. 7.1.8]. Since *T* is uniquely determined from *T*^∗^ and the knowledge of the root (that is the node labeled with *r*), we deduce that *φ*(*T*) singles out *T*. □

This result implies that the vectors of cophenetic values of *pairs of different taxa* single out unweighted phylogenetic trees without nested taxa.

#### Corollary 1

For every $T\;\in \;T_{n}$, let $\tilde{\varphi }(T)\;=\;\left(\varphi_{T}\right)$${\left(\left(i,j\right)\right)}_{1⩽i<j⩽n}\in R^{n(n-1)/2}$, with its elements lexicographically ordered in (*i*, *j*). Then, for every $T,T^{\prime }\in T_{n}$, if $\tilde{\varphi }(T)=\tilde{\varphi }(T^{\prime })$, then *T*=*T*^′^.

#### Proof

If *T* is unweighted and without nested taxa, then, for every taxon *i*,

$$\delta_{T}(i)=1+\max\{\varphi_{T}(i,j)|1⩽j⩽n,\;j\neq i\}$$

and therefore, in this case, *φ*(*T*) is uniquely determined by $\tilde{\varphi }(T)$. □

But in order to single out phylogenetic trees with non constant weights in the arcs or with nested taxa, it is necessary to take into account also the depths of the leaves. Actually, for example, there is no way to reconstruct from $\tilde{\varphi }(T)$ the weights of the pendant arcs: the depths of the leaves are needed. Or, without being able to compare depths with cophenetic values, there is no way to say whether a taxon is nested or not. More specifically, for instance, the three trees in Figure 1 have the same value of *φ*(1, 2), and hence the same vector $\tilde{\varphi }(T)$, but they are not isomorphic as weighted phylogenetic trees.

The cophenetic vector *φ*(*T*) of a weighted phylogenetic tree $T\in {WT}_{n}$ can be computed in optimal *O*(*n*^2^) time (assuming a constant cost for the addition of real numbers) by computing for each internal node *v*, its depth *δ*_*T*_(*v*) through a preorder traversal of *T*, and the pairs of taxa of which *v* is the LCA through a postorder traversal of the tree. Both preorder and postorder traversals are performed in linear time on the usual tree data structures.

### Cophenetic metrics

As we have seen in Theorem 1, the mapping

$$\varphi :{WT}_{n}\to R^{n(n+1)/2}$$

that sends each $T\in {WT}_{n}$ to its cophenetic vector *φ*(*T*), is injective up to isomorphism. As it is well known, this allows to induce metrics on ${WT}_{n}$ from metrics defined on powers of R. In particular, every *L*^*p*^ norm ∥ · ∥_*p*_ on $R^{n(n+1)/2}$, p⩾1, induces a *cophenetic metricd*_*φ*, *p*_ on ${WT}_{n}$ by means of

$$d_{\varphi ,p}(T_{1},T_{2})=∥\varphi (T_{1})-\varphi (T_{2}){∥}_{p},\;T_{1},T_{2}\in {WT}_{n}.$$

Recall that

$$∥(x_{1},\ldots ,x_{m}){∥}_{p}=\sqrt[{p}]{|x_{1}{|}^{p}+\cdots +|x_{m}{|}^{p}},$$

and so, for instance,

$$\begin{array}{l} d_{\varphi ,1}(T_{1},T_{2})=\underset{1⩽i⩽j⩽n}{\sum }|\varphi_{T_{1}}(i,j)-\varphi_{T_{2}}(i,j)|\\ d_{\varphi ,2}(T_{1},T_{2})=\sqrt{\underset{1⩽i⩽j⩽n}{\sum }{\left(\varphi_{T_{1}}(i,j)-\varphi_{T_{2}}(i,j)\right)}^{2}} \end{array}$$

are the cophenetic metrics on ${WT}_{n}$ induced by the Manhattan *L*^1^ and the euclidean *L*^2^ norms. One can also use Donoho’s *L*^0^ “norm” (which, actually, is not a proper norm)

$$∥(x_{1},\ldots ,x_{m}){∥}_{0}=\text{number of entries}\;x_{i}\;\text{that are}\;\neq 0$$

to induce a metric *d*_*φ*,0_(*T*_1_,*T*_2_) on ${WT}_{n}$, which turns out to be simply the Hamming distance between *φ*(*T*_1_) and *φ*(*T*_2_).

As we have seen in the previous subsection, the cophenetic vector of a phylogenetic tree in ${WT}_{n}$ can be computed in *O*(*n*^2^) time. For every $T_{1},T_{2}\in {WT}_{n}$, and assuming a constant cost for the addition and product of real numbers, the cost of computing *d*_*φ*,0_(*T*_1_, *T*_2_) (as the number of non-zero entries of *φ*(*T*_1_)-*φ*(*T*_2_)) is *O*(*n*^2^), and the cost of computing *d*_*φ*,*p*_(*T*_1_, *T*_2_)^*p*^, for p⩾1 (as the sum of the *p*-th powers of the entries of the difference *φ*(*T*_1_) - *φ*(*T*_2_)) is *O*(*n*^2^ + log2(*p*)*n*^2^), which is again *O*(*n*^2^) if we understand log(*p*) as part of the constant factor. Finally, the cost of computing *d*_*φ*,*p*_(*T*_1_, *T*_2_), p⩾1, as the *p*-th root of *d*_*φ*,*p*_(*T*_1_, *T*_2_)^*p*^ will depend on *p* and on the accuracy with which this root is computed. Assuming a constant cost for the computation of *p*-th roots with a given accuracy (notice that, in practice, for low *p* and accuracy, this step will be dominated by the computation of *d*_*φ*,*p*_(*T*_1_, *T*_2_)^*p*^), the total cost of computing *d*_*φ*,*p*_(*T*_1_, *T*_2_) is *O*(*n*^2^).

Next examples show some features of these cophenetic metrics.

#### Example 2

Let $T\in {UT}_{n}$, let (*u*, *v*) be an arc of *T* with *u* or *v* unlabeled, and let *T*^′^ be the phylogenetic tree in ${UT}_{n}$ obtained by *contracting* (*u*, *v*): that is, by removing the node *v* and the arc (*u*, *v*), labeling *u* with the label of *v* if it was labeled, and replacing every arc (*v*, *x*) in *T* by an arc (*u*, *x*). Notice that, in the passage from *T* to *T*^′^, for every *i*, *j* ∈ *S*: 

• If both *i,j* are descendants of *v* in *T*, then $\varphi_{T^{\prime }}(i,j)=\varphi_{T}(i,j)-1$.

• In any other case, $\varphi_{T^{\prime }}(i,j)=\varphi_{T}(i,j)$.

As a consequence,

$$\varphi_{T}(i,j)-\varphi_{T^{\prime }}(i,j)=\left\{\begin{array}{ll} 1 & \text{if}\;i,j≼v\\ 0 & \text{otherwise} \end{array}\right)$$

and therefore, if *n*_*v*_ is the number of descendant taxa of *v*,

dφ,0(T,T′)=nv+12,dφ,p(T,T′)=nv+12pifp⩾1.

So the contraction of an arc in an tree *T* (which is Robinson-Foulds’ *α*-operation [15]) yields a new tree *T*^′^ at a cophenetic distance from *T* that depends increasingly on the number of descendant taxa of the head of the contracted arc.

#### Example 3

Let $T_{0},T_{0}^{\prime }\in {WT}_{m}$, for some *m* < *n*, let $T\in {WT}_{n}$ be such that its subtree rooted at some node *z* is *T*_0_, and let $T^{\prime }\in {WT}_{n}$ be the tree obtained by replacing in *T* this subtree *T*_0_ by $T_{0}^{\prime }$.

Notice that, for every *i*, *j* ∈ {1, …, *n*}, $\varphi_{T}(i,j)=\delta_{T}(z)+\varphi_{T_{0}}(i,j)$ if *i*, *j* ⩽ *m*, and *φ*_*T*_(*i*, *j*) = *φ*_*T*_(*z*, *j*) if *i* ⩽ *m* and *j* > *m*, and the same holds in *T*^′^, replacing *T* and *T*_0_ by *T*^′^ and $T_{0}^{\prime }$, respectively. Since, moreover, $\delta_{T}(z)=\delta_{T^{\prime }}(z)$, $\varphi_{T}(z,j)=\varphi_{T^{\prime }}(z,j)$ for every *j* > *m*, and $\varphi_{T}(i,j)=\varphi_{T^{\prime }}(i,j)$ for every *i*,*j* > *m*, we conclude that

$$\varphi (T)-\varphi (T^{\prime })=\varphi (T_{0})-\varphi (T_{0}^{\prime })$$

and hence

$$d_{\varphi ,p}(T,T^{\prime })=d_{\varphi ,p}(T_{0},T_{0}^{\prime }).$$

So, the cophenetic metrics are local, as other popular metrics like the Robinson Foulds or the triples metrics, but unlike other popular metrics, like for instance the nodal metrics.

## Results and discussion

### Minimum and maximum values for cophenetic metrics

Our first goal is to find the smallest non-negative value of *d*_*φ*,*p*_ on several spaces of phylogenetic trees, and the pairs of trees at which it is reached. These pairs of trees at minimum distance can be understood as ‘adjacent’ in the corresponding metric space, and their characterization yields a first step towards understanding how cophenetic metrics measure the difference between two trees.

Notice that this problem makes no sense for weighted phylogenetic trees. For instance, if we add or subtract an *ϵ* > 0 to the weight of a pendant arc in a tree *T*, without changing its topology, the distance between *T* and the resulting tree will be *ϵ*, which can be as small as desired. So, we only consider this problem on ${UT}_{n}$, $T_{n}$, and ${BT}_{n}$.

In order to simplify the statements, set

$$D_{p}(T_{1},T_{2})=\left\{\begin{array}{ll} d_{\varphi ,0}(T_{1},T_{2}) & \text{if}\;p=0\\ d_{\varphi ,p}{\left(T_{1},T_{2}\right)}^{p} & \text{if}\;p⩾1 \end{array}\right)$$

The following easy result, which is a direct consequence of the fact that $D_{p}(T_{1},T_{2})⩾D_{0}(T_{1},T_{2})$ for every p⩾1 and $T_{1},T_{2}\in {UT}_{n}$, will be used in the proof of the next propositions.

#### Lemma 1

Assume that, for every pair of different trees *T*_1_, *T*_2_ in ${UT}_{n}$, $T_{n}$ or ${BT}_{n}$ such that *D*_0_(*T*_1_, *T*_2_) is minimum on this space, we have that *D*_*p*_(*T*_1_, *T*_2_) = *D*_0_(*T*_1_,*T*_2_). Then, the minimum non-zero value of *D*_*p*_ on this space of trees is equal to the minimum non-zero value of *D*_0_ on it, and it is reached at exactly the same pairs of trees.

The least non-negative values of *D*_*p*_, for *p* ∈ {0}∪[1, *∞*[, on ${UT}_{n}$, $T_{n}$, and ${BT}_{n}$, together with an explicit description of the pairs of trees where these minimum values are reached, are given by the next three propositions. We give their proofs in the Additional file 1.

#### Proposition 1

The minimum non-negative value of *D*_*p*_ on ${UT}_{n}$, for *p* ∈ {0}∪[1, *∞*[ and n⩾2, is 1. And for every $T,T^{\prime }\in {UT}_{n}$, *D*_*p*_(*T*, *T*^′^) = 1 if, and only if, one of them is obtained from the other by contracting a pendant arc.

So, not every tree in ${UT}_{n}$ has neighbors at cophenetic distance 1: only those trees with some leaf whose parent is unlabeled. Now, it is not difficult to check that a tree $T\in {UT}_{n}$ such that all its leaves have labeled parents has some tree *T*^′^ such that *D*_*p*_(*T*, *T*^′^) = 2, which is the minimum value of *D*_*p*_ on ${UT}_{n}$ greater than 1. One such *T*^′^ is obtained by choosing a pendant arc in *T* and interchanging the labels of its source and its target nodes.

#### Proposition 2

**Figure 3:**
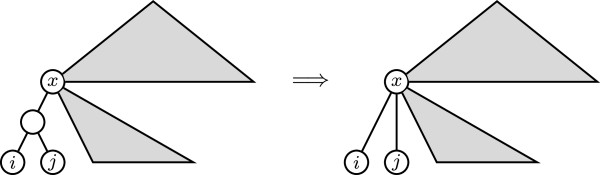
Contraction of an arc ending in the parent of a cherry.

**Figure 4:**
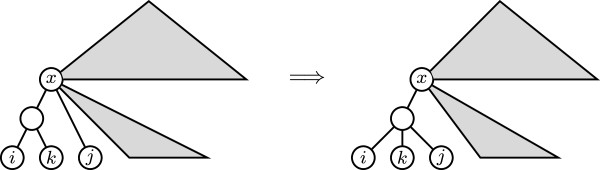
Pruning and regrafting an uncle of a cherry to make it a sibling of them.

The minimum non-negative value of *D*_*p*_ on $T_{n}$, for *p* ∈ {0}∪[1, *∞* [ and n⩾3, is 3. And for every $T,T^{\prime }\in T_{n}$, *D*_*p*_(*T*, *T*^′^) = 3 if, and only if, one of them is obtained from the other by means of one of the following two operations: 

(a) Contracting an arc ending in the parent of a cherry (see Figure 3)

(b) Pruning and regrafting a leaf that is a sibling of the root of a cherry, to make it a sibling of the leaves in the cherry (see Figure 4)

So, every tree $T\in T_{n}$ has neighbors *T*^′^ such that *D*_*p*_(*T*, *T*^′^) = 3. Indeed, take an internal node *v* in *T* of largest depth, so that all its children are leaves. If *v* has exactly two children, one such neighbor of *T* is obtained by contracting the arc ending in *v*. If *v* has more than two children, one such neighbor of *T* is obtained by replacing any two children of *v* by a cherry (that is, taking two children *i, j* of *v*, removing the arcs (*v*, *i*) and (*v*, *j*), and then adding a new node *v*_0_ and arcs (*v*, *v*_0_), (*v*_0_, *i*), and (*v*_0_, *j*)).

#### Proposition 3

**Figure 5:**
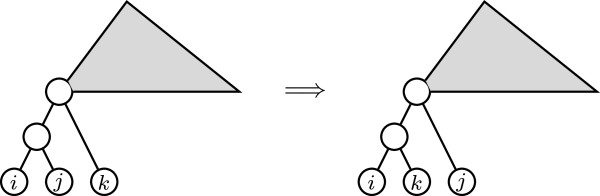
Reorganizing a triplet.

**Figure 6:**
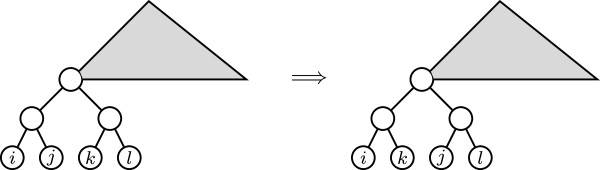
Reorganizing a completely branched quartet.

The minimum non-negative value of *D*_*p*_ on ${BT}_{n}$, for *p* ∈ {0}∪ [1, *∞*[ and n⩾3, is 4. And for every $T,T^{\prime }\in {BT}_{n}$, *D*_*p*_(*T*, *T*^′^) = 4 if, and only if, one of them is obtained from the other by means of one of the following operations: 

(a) Reorganizing a triplet (see Figure 5)

(b) Reorganizing a completely branched quartet (see Figure 6)

So again, every tree $T\in {BT}_{n}$ has neighbors *T*^′^ such that *D*_*p*_(*T*, *T*^′^) = 4. Indeed, take an internal node *v* in *T* of largest depth, so that its two children are leaves. Let *w* be the parent of *v*. Then, either the other child of *w* is a leaf, in which case *w* is the root of a triple and reorganizing its taxa we obtain a neighbor of *T*, or the other child of *w* is the parent of a cherry (it will have the same, maximum, depth as *v*), in which case *w* is the root of a completely branched quartet and reorganizing its taxa we obtain a neighbor of *T*.

We focus now on the diameter, that is, the largest value of *d*_*φ*,*p*_ on the spaces of unweighted phylogenetic trees (as in the case of the minimum non-zero value, and for the same reasons, the problem of finding the diameter makes no sense for weighted trees). Unfortunately, we have not been able to find exact formulas for it, but we have obtained its order, which we give in the next proposition. We also give its proof in the Additional file 1.

#### Proposition 4

The diameter of *d*_*φ*,*p*_ on ${UT}_{n}$, $T_{n}$, and ${BT}_{n}$ is in *Λ*(*n*^2^) if *p* = 0 and in *Λ*(*n*^(*p* + 2) / *p*^) if p⩾1.

In particular, the diameter of *d*_*φ*,1_ on these spaces is in *Λ*(*n*^3^), and the diameter of *d*_*φ*,2_ is in *Λ*(*n*^2^).

### Numerical experiments

We have performed several numerical experiments concerning the distributions of *d*_*φ*,1_ and *d*_*φ*,2_, and the correlation of these metrics with other phylogenetic tree comparison metrics. The results of all these experiments can be found in the web page http://bioinfo.uib.es/âˆ¼recerca/phylotrees/cophidist/. In this section we report only on some significant results obtained through these experiments.

As a first experiment, we have generated all trees in ${BT}_{n}$ and $T_{n}$, for *n* = 3, 4, 5, 6, and for all pairs of them we have computed: 

• The cophenetic distances *d*_*φ*,1_ and *d*_*φ*,2_ on ${BT}_{n}$ and $T_{n}$.

• The Robinson-Foulds distance *d*_RF_ on ${BT}_{n}$ and $T_{n}$[15].

• The classical nodal distances *d*_nodal,1_ and *d*_nodal,2_ on ${BT}_{n}$, which compare the vectors of distances between pairs of taxa by means of the Manhattan and the Euclidean norms, respectively; see [5] and [18], respectively, as well as [20].

• The splitted nodal distances $d_{\text{nodal},1}^{\text{sp}}$ and $d_{\text{nodal},2}^{\text{sp}}$ on $T_{n}$, which compare the matrices of splitted path lengths between pairs of taxa by means of the Manhattan and the Euclidean norms, respectively; see [20].

**Figure 7:**
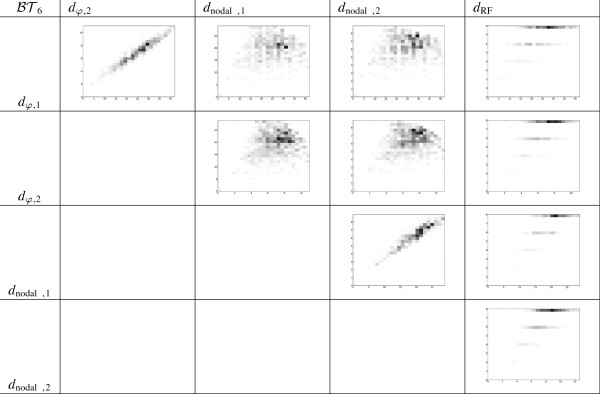
**2D-histograms showing the relationship between different distances on**${BT}_{6}$**.**

**Figure 8:**
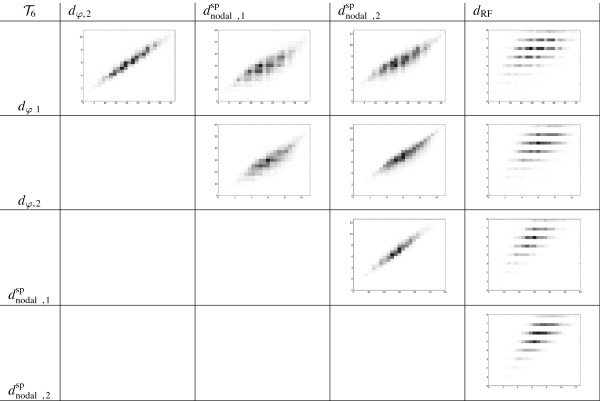
**2D-histograms showing the relationship between different distances on**$T_{6}$**.**

In order to analyze this data, we have plotted 2D-histograms for all pairs of metrics and we have computed their Spearman’s rank correlation coefficient. On the one hand, the 2D-histograms for ${BT}_{6}$ and $T_{6}$ (the most significative case) are given in Figures 7 and 8, respectively. For each pair of distances, we have divided the range of values that each of the distances gets into 25 subranges, and computed how many pairs of trees fall into each of the 25 × 25 different possibilities. Each of these possibilities is represented by a rectangle in a grid, whose darkness level is proportional of the number of trees. On the other hand, the Spearman’s rank correlation coefficient between the aforementioned distances in the most significative case of *n* = 6 are given in Tables 2 and 3.

**Table 2:** Spearman’s rank correlation coefficient between different distances on ${BT}_{6}$

${BT}_{6}$	***d***_***φ*,2**_	***d***_**nodal,1**_	***d***_**nodal,2**_	***d***_**RF**_
*d*_*φ*,1_	0.966309	0.066217	0.057751	0.473775
*d*_*φ*,2_		0.093708	0.100914	0.501130
*d*_nodal,1_			0.928421	0.585127
*d*_nodal,2_				0.623644

**Table 3:** Spearman’s rank correlation coefficient between different distances on $T_{6}$

$T_{6}$	***d***_***φ*,2**_	$d_{\text{nodal},1}^{\text{sp}}$	$d_{\text{nodal},2}^{\text{sp}}$	***d***_**RF**_
*d*_*φ*,1_	0.965115	0.803159	0.864113	0.505631
*d*_*φ*,2_		0.831387	0.902573	0.529837
$d_{\text{nodal},1}^{\text{sp}}$			0.957057	0.665752
$d_{\text{nodal},2}^{\text{sp}}$				0.642203

These histograms and tables show that *d*_*φ*,1_ and *d*_*φ*,2_ are highly correlated, and that each *d*_*φ*,*i*_, *i* = 1, 2, is highly correlated with the corresponding $d_{\text{nodal},i}^{\text{sp}}$ on $T_{6}$. This is not a surprise, because both types of metrics are based on encodings of phylogenetic trees related to the position in the tree of the LCA of every pair of leaves: remember the relationship between depths, cophenetic values and splitted path lengths recalled in the Background section. More surprising to us is the low correlation between each *d*_*φ*,*i*_, and the corresponding *d*_nodal,*i*_ on ${BT}_{6}$, because of the relationship between depths, cophenetic values and patristic distances also recalled in the Background section. The very low correlation between the cophenetic metrics and the Robinson-Foulds metric simply shows that these metrics measure different notions of similarity.

**Figure 9:**
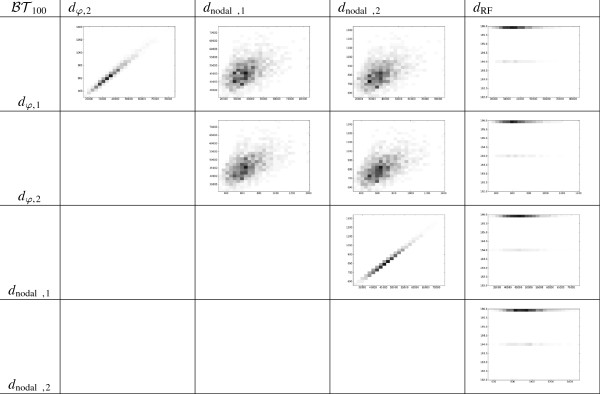
**2D-histograms showing the relationship between different distances on**${BT}_{100}$**.**

**Figure 10:**
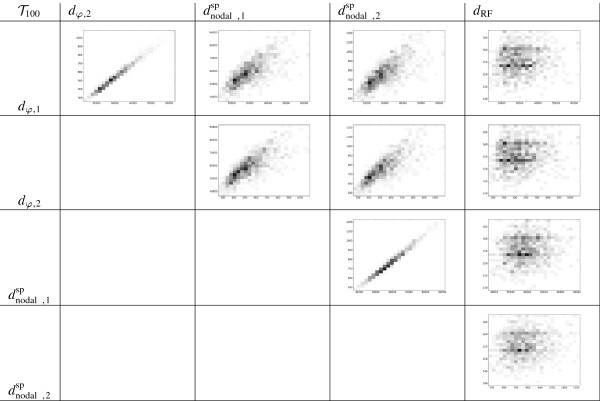
**2D-histograms showing the relationship between different distances on**$T_{100}$**.**

Our second experiment is for values of *n* greater than 6. The numbers of trees in each of the spaces $T_{n}$ and ${BT}_{n}$ make it unfeasible to compute the distances between all pairs of trees. Hence, we have randomly and uniformly generated pairs of trees in each of these spaces for n=10,20,…,100 until the approximated value of the Spearman’s rank correlations of all pairs of distances converge up to 3 significant digits. The corresponding 2D-histograms and Spearman’s rank correlation coefficient tables for the most significative case of *n* = 100 are shown in Figures 9 and 10 and Tables 4 and 5. These diagrams and tables confirm the very high correlation between *d*_*φ*,1_ and *d*_*φ*,2_, and very low correlation of these metrics and the nodal and Robinson-Foulds metrics. The correlation between each *d*_*φ*,*i*_, *i* = 1, 2, and the corresponding $d_{\text{nodal},i}^{\text{sp}}$ is still significant, but it decreases as *n* increases.

**Table 4:** Spearman’s rank correlation coefficient between different distances on ${BT}_{100}$

${BT}_{100}$	***d***_***φ*,2**_	***d***_**nodal,1**_	***d***_**nodal,2**_	***d***_**RF**_
*d*_*φ*,1_	0.986933	0.447140	0.448265	-0.00080
*d*_*φ*,2_		0.513306	0.514363	0.003281
*d*_nodal,1_			0.998478	0.012643
*d*_nodal,2_				0.012391

**Table 5:** Spearman’s rank correlation coefficient between different distances on $T_{100}$

$T_{100}$	***d***_***φ*,2**_	$d_{\text{nodal},1}^{\text{sp}}$	$d_{\text{nodal},2}^{\text{sp}}$	***d***_**RF**_
*d*_*φ*,1_	0.987184	0.731755	0.753918	0.091556
*d*_*φ*,2_		0.780030	0.803423	0.088390
$d_{\text{nodal},1}^{\text{sp}}$			0.990944	0.132030
$d_{\text{nodal},2}^{\text{sp}}$				0.118336

**Figure 11:**
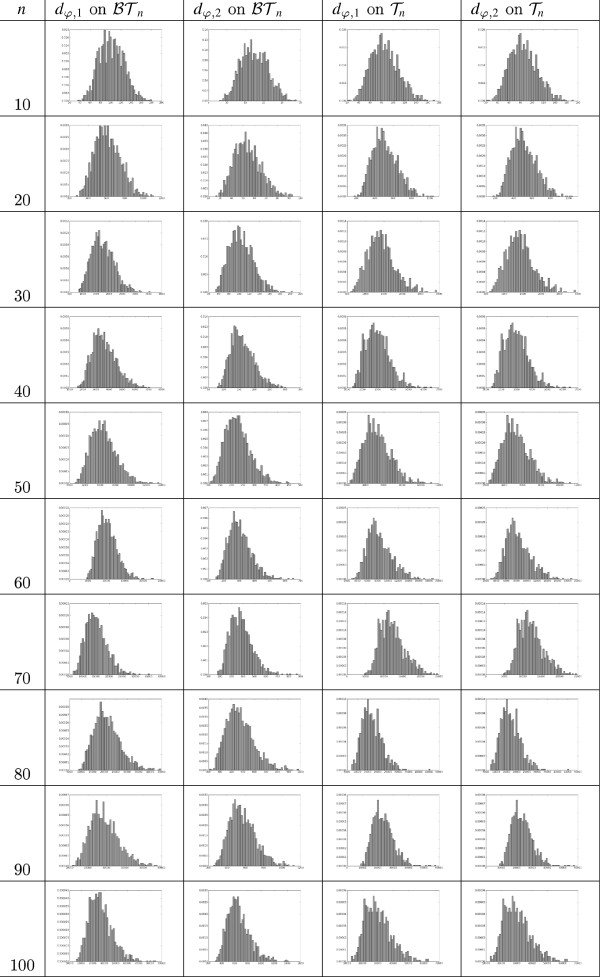
**Histograms of the distributions of *d***_***φ,1 ***_**and *d***_***φ,2 ***_**on**$T_{n}$**and**${BT}_{n}$**for**n=10,20,…,100**.**

Finally, in Figure 11 we have plotted the histograms of the distributions of *d*_*φ*,1_ and *d*_*φ*,2_ on ${BT}_{n}$ and $T_{n}$ for n=10,20,…,100. As it can be seen, they are positive skewed, like the splitted nodal metrics [20], Figure 5], but unlike other metrics like the Robinson-Foulds [32] or the transposition distance [33], Figure 2], which are negative skewed, or the triples metric [16], which is approximately normal.

## Conclusions

Following a fifty years old idea of Sokal and Rohlf [4], we have encoded a weighted phylogenetic tree with nested taxa by means of its vector of cophenetic values of pairs of taxa, adding moreover to this vector the depths of single taxa. These positive real-valued vectors single out weighted phylogenetic trees with nested taxa, and therefore they can be used to define metrics to compare such trees. We have defined a family of metrics *d*_*φ*,*p*_, for *p* ∈ {0}∪[1, *∞*[, by comparing these vectors through the *L*^*p*^ norm.

We cannot advocate the use of any cophenetic metric *d*_*φ*,*p*_ over the other ones except, perhaps, warning against the use of the Hamming distance *d*_*φ*,0_ because it is too uninformative. Since the most popular norms on $R^{m}$ are the Manhattan *L*^1^ and the Euclidean *L*^2^, it seems natural to use *d*_*φ*,1_ or *d*_*φ*,2_. And since these two metrics are very highly correlated, the comparison of trees using one or the other will not differ greatly. Each one of these metrics has its own advantages.

On the one hand, the computation of *d*_*φ*,1_ does not involve roots, and therefore it can be computed exactly. Moreover, it takes integer values on unweighted trees and in this case its range of values is greater, thus being more discriminative. Actually, since ∥*x*∥_*p*_ ⩽ ∥*x*∥_1_ for every $x\in R^{m}$ and p⩾1, we have that

$$d_{\varphi ,p}(T_{1},T_{2})\leq d_{\varphi ,1}(T_{1},T_{2})\;\text{for every}T_{1},T_{2}\in {WT}_{n}.$$

On the other hand, the comparison of cophenetic vectors by means of the Euclidean norm enables the use of many geometric and clustering methods that are not available otherwise. In particular, it is possible to compute the mean value of the square of *d*_*φ*,2_ under different evolutionary models. We shall report on this elsewhere.

As a rule of thumb, and as we already advised in the context of splitted nodal metrics [20], we suggest using *d*_*φ*,1_ when the trees are unweighted, because these trees can be seen as discrete objects and thus their comparison through a discrete tool as the Manhattan norm seems appropriate. When the trees have arbitrary positive real weights, they should be understood as belonging to a continuous space [34], and then the Euclidean norm is more appropriate.

Future work will include a deeper study of the distribution of *d*_*φ*,1_ and *d*_*φ*,2_ on different spaces of unweighted phylogenetic trees.

## Endnotes

^a^There are some details to be filled here, because for technical reasons we shall allow the root of our phylogenetic trees to have out-degree 1 without being labeled, and this case is not covered by [20, Thm. 10], but it is not difficult to modify the argument given above to cover also this case.^b^http://en.wikipedia.org/wiki/Phylogenetic_tree

## Competing interests

The authors declare that they have no competing interests.

## Authors’ contributions

AM and FR developed the theoretical part of the paper. GC, LR and DS implemented the algorithms and performed the numerical experiments. GC and DS prepared the Additional file 1 web page. FR prepared the first version of the manuscript. All authors revised, discussed, and amended the manuscript and approved its final version. All authors read and approved the final manuscript.

## Supplementary Material

Additional file 1 Proofs of propositions 1–4. Click here for file
