# Supplementary material for: Cophenetic metrics for phylogenetic trees, after Sokal and Rohlf
Source: BMC Bioinformatics. 2013 Jan 16;14:3. doi: 10.1186/1471-2105-14-3 (PMC3716993; doi:10.1186/1471-2105-14-3)
Supplement: Additional file 1 — Proofs of propositions 1–4. [file 1471-2105-14-3-S1.pdf]

Cophenetic metrics for phylogenetic trees, after Sokal and Rohlf (Supplementary Material)

Gabriel Cardona, Arnau Mir, Francesc Rosselló*, Lucía Rotger, and David Sánchez

Department of Mathematics and Computer Science, University of the Balearic Islands, E-07122 Palma de Mallorca, Spain

Email: Gabriel Cardona - gabriel.cardona@uib.es; Arnau Mir - arnau.mir@uib.es; Francesc Rosselló - cesc.rossello@uib.es; Lucía Rotger - lucia.rotger@uib.es; David Sánchez - dscharles@gmail.com;

*Corresponding author

Proofs of Propositions 1–4

Proof of Proposition 1

By Lemma 1, it is enough to prove that the minimum non-zero value of D_0 is 1, and that all pairs $T, T' \in \mathcal{UT}_n$ such that $D_0(T,T') = 1$ also satisfy that $D_p(T,T') = 1$ for every $p \geqslant 1$.

As we have seen in Example 2, if we contract a pendant arc in a tree T, we obtain a new tree T' such that $D_p(T,T') = 1$, for every $p \in \{0\} \cup [1, \infty[$, and this is of course the smallest possible non-negative value of D_p on $\mathcal{UT}_n$. It remains to prove that this is the only way we can obtain a pair of trees such that $D_0(T,T') = 1$.

So, let $T, T' \in \mathcal{UT}_n$ be such that $\varphi(T) = \varphi(T') + m \cdot e_{i,j}$ for some $m \geqslant 1$ and $1 \leqslant i, j \leqslant n$ (where $e_{i,j}$ stands for the vector of length $n(n+1)/2$ with all entries 0 except an 1 in the entry corresponding to the pair (i,j)); that is, T and T' are such that $\varphi_T(i,j) = \varphi_{T'}(i,j) + m$, for some $m \geqslant 1$, and $\varphi_T(x,y) = \varphi_{T'}(x,y)$ for every $(x,y) \neq (i,j)$. Let us prove first of all that $m = 1$. So, assume that $m \geqslant 2$ and let us reach a contradiction.

Since $\varphi_T(i,j) > 0$, there exists some taxon $k \neq i,j$ that is a descendant in T of the parent of $[i,j]_T$. In other words, such that $[i,k]_T = [j,k]_T$ is the parent of $[i,j]_T$. But then

$$\varphi_{T'}(i,k) = \varphi_T(i,k) = \varphi_T(i,j) - 1 = \varphi_{T'}(i,j) + (m-1) > \varphi_{T'}(i,j)$$
$$\varphi_{T'}(j,k) = \varphi_T(j,k) = \varphi_T(i,j) - 1 = \varphi_{T'}(i,j) + (m-1) > \varphi_{T'}(i,j)$$

which cannot hold simultaneously: if $\varphi_{T'}(i,k) > \varphi_{T'}(i,j)$, then $\varphi_{T'}(j,k) = \varphi_{T'}(i,j)$. This shows that $m = 1$, and thus $\varphi(T) = \varphi(T') + e_{i,j}$.

Let us prove now that it cannot happen that $i \neq j$. Indeed, assume that $i \neq j$. If $\varphi_{T'}(i,j) = \delta_{T'}(i)$, then

$$\varphi_T(i,j) = \varphi_{T'}(i,j) + 1 = \delta_{T'}(i) + 1 = \delta_T(i) + 1,$$

which is impossible. This implies that $\varphi_{T'}(i,j) < \delta_{T'}(i), \delta_{T'}(j)$. If, now, $\varphi_{T'}(i,j) < \delta_{T'}(i) - 1$, then there will exist some leaf k such that $[i,k]_{T'}$ is the child of $[i,j]_{T'}$ in the path from $[i,j]_{T'}$ to i. Then $\varphi_{T'}(i,k) = \varphi_{T'}(i,j) + 1$ and $\varphi_{T'}(j,k) = \varphi_{T'}(i,j)$, which entail that

$$\varphi_T(i,k) = \varphi_{T'}(i,k) = \varphi_{T'}(i,j) + 1 = \varphi_T(i,j) > \varphi_{T'}(i,j) = \varphi_{T'}(j,k) = \varphi_T(j,k),$$

which is also impossible. So, if $i \neq j$, the only possibility is that $\varphi_{T'}(i,j) = \delta_{T'}(i) - 1 = \delta_{T'}(j) - 1$, but then it would imply that $\varphi_T(i,j) = \varphi_{T'}(i,j) + 1 = \delta_T(i) = \delta_T(j)$ and hence that $[i,j]_T = i = j$, which is again impossible.

So, if $\varphi(T) = \varphi(T') + e_{i,j}$ then it must happen that $i = j$. In this case, moreover, i must be a leaf in T with unlabeled parent. Indeed, if i is not a leaf, then there is some leaf k such that $i = [i,k]_T$ and hence $\delta_T(i) = \varphi_T(i,k)$. Then, $\delta_{T'}(i) = \delta_T(i) - 1 = \varphi_T(i,k) - 1 = \varphi_{T'}(i,k) - 1$, which is impossible. So, i is a leaf in T. And if its parent is labeled, say with l, then $\delta_T(i) = \delta_T(l) + 1$ and $\delta_T(l) = \varphi_T(i,l)$. Thus, in T', $\delta_{T'}(i) = \delta_T(i) - 1 = \delta_T(l) = \delta_{T'}(l)$ and $\delta_{T'}(i) = \delta_T(l) = \varphi_T(i,l) = \varphi_{T'}(i,l)$, which is also impossible, since it would imply that $[i,l]_{T'} = i = l$.

So, finally, it must happen that i is a leaf in T and its parent is not labeled. Let T_0 be the phylogenetic tree obtained from T by contracting the pendant arc ending in i. Then $\varphi(T_0) = \varphi(T) - e_{i,i} = \varphi(T')$, and this implies, by Theorem 1, that $T_0 = T'$.

This finishes the proof that the only pairs $T, T' \in \mathcal{WT}_n$ such that $D_0(T,T') = 1$ are those where one of them is obtained from the other by the contraction of a pendant arc. Since these pairs of trees also satisfy that $D_p(T,T') = 1$ for every $p \geqslant 1$, this completes the proof of the proposition. □

Proof of Proposition 2

To ease the task of the reader, we split this proof into several lemmas. To begin with, notice that there are pairs of trees $T, T' \in \mathcal{T}_n$ such that $D_p(T,T') = 3$ for every $p \in \{0\} \cup [1,\infty[$: for instance, by Example 2, when T' is obtained from T by contracting an arc ending in the root of a cherry. So, the minimum non-zero value of $D_p(T,T')$ on $\mathcal{T}_n$ is at most 3.

Lemma 1. *If $T, T' \in \mathcal{T}_n$ are such that $D_0(T,T') > 0$, then there exists a pair of different taxa $i \neq j$ such that $\varphi_T(i,j) \neq \varphi_{T'}(i,j)$.*

Proof. If $\varphi_T(i,j) = \varphi_{T'}(i,j)$ for every $i \neq j$, then, by Corollary 1, $T = T'$ and therefore $D_0(T,T') = 0$. □

So, every pair of phylogenetic trees in $\mathcal{T}_n$ at non-zero D_0 distance must have a pair of different leaves with different cophenetic values.

Lemma 2. *Let $T, T' \in \mathcal{T}_n$ be such that $\varphi_T(i,j) = \varphi_{T'}(i,j) + m$, for some $1 \leqslant i < j \leqslant n$ and some $m \geqslant 1$. Let $k \neq i,j$ be a leaf such that there exists a path from $[i,j]_{T'}$ to $[i,k]_{T'}$ of length l, for some $l \geqslant 1$. Then:*

(a) If $\varphi_T(i,k) = \varphi_{T'}(i,k)$, then $\varphi_T(j,k) \geqslant \varphi_{T'}(j,k) + \min\{m,l\}$

(b) If $\varphi_T(j,k) = \varphi_{T'}(j,k)$, then $\varphi_T(i,k) = \varphi_{T'}(i,k) - l$

Proof. From the assumptions we have that $\varphi_{T'}(i,k) = \varphi_{T'}(i,j) + l = \varphi_{T'}(j,k) + l$. Now:

(a) Assume that $\varphi_T(i,k) = \varphi_{T'}(i,k)$. Then,

$$\varphi_T(i,k) = \varphi_{T'}(i,k) = \varphi_{T'}(i,j) + l = \varphi_T(i,j) - (m-l),$$

and then

- If $m > l$, then $\varphi_T(i,k) < \varphi_T(i,j)$, that is, $[i,j]_T \prec [i,k]_T$, and thus

$$\varphi_T(j,k) = \varphi_T(i,k) = \varphi_{T'}(i,k) = \varphi_{T'}(j,k) + l$$

- If $m = l$, then $\varphi_T(i,k) = \varphi_T(i,j)$, that is, $[i,k]_T = [i,j]_T$, and thus

$$\varphi_T(j,k) \geqslant \varphi_T(i,j) = \varphi_{T'}(i,j) + m = \varphi_{T'}(j,k) + m$$

- If $m < l$, then $\varphi_T(i,k) > \varphi_T(i,j)$, that is, $[i,k]_T \prec [i,j]_T$, and thus

$$\varphi_T(j,k) = \varphi_T(i,j) = \varphi_{T'}(i,j) + m = \varphi_{T'}(j,k) + m$$

(b) Assume that $\varphi_T(j,k) = \varphi_{T'}(j,k)$. Then

$$\varphi_T(j,k) = \varphi_{T'}(j,k) = \varphi_{T'}(i,j) = \varphi_T(i,j) - m,$$

so that $[i,j]_T \prec [j,k]_T$, and thus

$$\varphi_T(i,k) = \varphi_T(j,k) = \varphi_{T'}(j,k) = \varphi_{T'}(i,j) = \varphi_{T'}(i,k) - l$$

□

As a direct consequence of this lemma we obtain the following result.

Corollary 1. *Let $T, T' \in \mathcal{T}_n$ be such that $\varphi_T(i,j) = \varphi_{T'}(i,j) + m$, for some $1 \leqslant i < j \leqslant n$ and some $m \geqslant 1$. Let N be the number of leaves k such that $k \neq i,j$ and either $[i,k]_{T'} \prec [i,j]_{T'}$ or $[j,k]_{T'} \prec [i,j]_{T'}$. Then,*

$$D_0(T,T') \geqslant N + 1.$$

□

Lemma 3. *Let $T, T' \in \mathcal{T}_n$ be such that $D_0(T, T') \leqslant 3$. If $\varphi_T(i,j) = \varphi_{T'}(i,j) + m$, for some $1 \leqslant i < j \leqslant n$ and some $m \geqslant 1$, then $m = 1$.*

Proof. If $\delta_{T'}(i) = \delta_T(i)$, then $\delta_{T'}(i) = \delta_T(i) > \varphi_T(i,j) = \varphi_{T'}(i,j) + m$ which implies that there are at least m leaves k such that $[i,k]_{T'} \prec [i,j]_{T'}$. Then, by the last corollary, $D_0(T,T') \geqslant m+1$. Now, if $\delta_{T'}(j) = \delta_T(j)$, then for the same reason there are at least m leaves k such that $[j,k]_{T'} \prec [i,j]_{T'}$ and they increase $D_0(T,T')$ to at least $2m+1$, while if $\delta_{T'}(j) \neq \delta_T(j)$, then $D_0(T,T') \geqslant m+2$. We conclude then that if $\delta_{T'}(i) = \delta_T(i)$, then $m = 1$. By symmetry, if $\delta_{T'}(j) = \delta_T(j)$, then $m = 1$, either.

Finally, if $\delta_{T'}(i) \neq \delta_T(i)$ and $\delta_{T'}(j) \neq \delta_T(j)$, and since $\varphi_T(i,j) \neq \varphi_{T'}(i,j)$, we have that $\varphi_T(x,y) = \varphi_{T'}(x,y)$ for every $(x,y) \neq (i,i),(j,j),(i,j)$. Let now $k \neq i,j$ be a taxon such that $[i,k]_T = [j,k]_T$ is the parent of $[i,j]_T$ in T. Then

$$\varphi_{T'}(i,k) = \varphi_T(i,k) = \varphi_T(i,j) - 1 = \varphi_{T'}(i,j) + (m-1)$$

and therefore, if $m \geqslant 2$, $\varphi_{T'}(i,k) > \varphi_{T'}(i,j)$ and then, by Lemma 2, either $\varphi_T(i,k) \neq \varphi_{T'}(i,k)$ or $\varphi_T(j,k) \neq \varphi_{T'}(j,k)$, which, as we have seen, is impossible. Thus, $m = 1$ in all cases. □

Lemma 4. *Let $T, T' \in \mathcal{T}_n$ be such that $D_0(T,T') \leqslant 3$. If $\varphi_T(i,j) = \varphi_{T'}(i,j) + 1$, for some $1 \leqslant i < j \leqslant n$, then $(\delta_{T'}(i) - \varphi_{T'}(i,j)) + (\delta_{T'}(j) - \varphi_{T'}(i,j)) \leqslant 3$.*

Proof. Let us assume that $(\delta_{T'}(i) - \varphi_{T'}(i,j)) + (\delta_{T'}(j) - \varphi_{T'}(i,j)) \geqslant 4$ and let us reach a contradiction.

Assume first that $\delta_{T'}(i) \geqslant \varphi_{T'}(i,j) + 3$. Then, there are at least two leaves k_1, k_2 such that $[i,k_1]_{T'}, [i,k_2]_{T'} \prec [i,j]_{T'}$. Since each such leaf contributes at least 1 to $D_0(T,T') \leqslant 3$, we conclude that there must be exactly two such leaves and, moreover, $\varphi_T(x,y) = \varphi_{T'}(x,y)$ for every $(x,y) \neq (i,j),(i,k_1),(j,k_1),(i,k_2),(j,k_2)$. But then, on the one hand, $\delta_T(j) = \delta_{T'}(j)$ and, on the other hand, $\delta_{T'}(j) = \varphi_{T'}(i,j) + 1$ (otherwise, there would be some other leaf k such that $[j,k]_{T'} \prec [i,j]_{T'}$, which, by Lemma 2 would satisfy that $\varphi_T(i,k) \neq \varphi_{T'}(i,k)$ or $\varphi_T(j,k) \neq \varphi_{T'}(j,k)$). Combining these two equalities we obtain $\delta_T(j) = \varphi_T(i,j)$, which is impossible in a tree without nested taxa. This proves that $\delta_{T'}(i) \leqslant \varphi_{T'}(i,j) + 2$ and, by symmetry, that $\delta_{T'}(j) \leqslant \varphi_{T'}(i,j) + 2$, as we claimed.

Thus, it remains to prove that the case $\delta_{T'}(i) = \delta_{T'}(j) = \varphi_{T'}(i,j) + 2$ is impossible. So, assume this case holds, and let's reach a contradiction. By Corollary 1, if $D_0(T,T') \leqslant 3$ and $\delta_{T'}(i) = \delta_{T'}(j) = \varphi_{T'}(i,j) + 2$, then there can exist only one extra leaf k pending from the parent of i and one extra leaf l pending from the parent of j: see Fig. 1, where the grey triangle stands for the (possibly empty) subtree consisting of all other descendants of $[i,j]_{T'}$. Moreover, since $\varphi_T(i,j) = \varphi_{T'}(i,j) + 1$ and since both k and l contribute at least 1

to $D_0(T,T') \leqslant 3$, we conclude that $\varphi_T(x,y) = \varphi_{T'}(x,y)$ for every $(x,y) \neq (i,j),(i,k),(j,k),(i,l),(j,l)$. In particular

$$\begin{array}{l}\varphi_T(k,l) = \varphi_{T'}(k,l) = \varphi_{T'}(i,j) = \varphi_T(i,j) - 1\\ \delta_T(i) = \delta_{T'}(i) = \varphi_{T'}(i,j) + 2 = \varphi_T(i,j) + 1\\ \delta_T(j) = \delta_T(k) = \delta_T(l) = \varphi_T(i,j) + 1 \text{ for the same reason}\end{array}$$

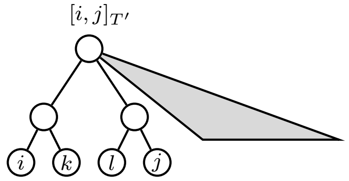

Figure 1: The subtree of T' rooted at $[i,j]_{T'}$ in the proof of Lemma 4.

Now we shall prove that, in this situation, each one of k,l contributes actually at least 2 to $D_0(T,T')$, and therefore $D_0(T,T') \geqslant 5$, which contradicts the assumption that $D_0(T,T') \leqslant 3$.

(1) Assume that $\varphi_T(i,k) = \varphi_{T'}(i,k)$. Then, by Lemmas 2.(a) and 3, $\varphi_T(j,k) = \varphi_{T'}(j,k) + 1$, and hence

$$\begin{array}{l}\varphi_T(i,k) = \varphi_{T'}(i,k) = \varphi_{T'}(j,k) + 1 = \varphi_T(j,k)\\ \varphi_T(i,k) = \varphi_{T'}(i,k) = \varphi_{T'}(i,j) + 1 = \varphi_T(i,j)\\ \delta_T(i) = \delta_T(j) = \delta_T(k) = \delta_T(l) = \varphi_T(i,j) + 1\\ \varphi_T(k,l) = \varphi_T(i,j) - 1\end{array}$$

Thus, the subtree of T rooted at $[k,l]_T$ contains a subtree of the form described in Fig. 2, for at least one leaf h. But then

$$\varphi_{T'}(l,h) = \varphi_T(l,h) = \varphi_T(i,j) = \varphi_{T'}(i,j) + 1 = \varphi_{T'}(l,j)$$

which is impossible, since it would imply that h is another descendant of $[l,j]_{T'}$. Therefore, $\varphi_T(i,k) \neq \varphi_{T'}(i,k)$ and, by symmetry, $\varphi_T(j,l) \neq \varphi_{T'}(j,l)$.

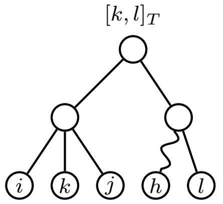

Figure 2: A subtree of the subtree of T rooted at $[k,l]_T$ in case (1) in the proof of Lemma 4.

(2) Assume now that $\varphi_T(i,l) = \varphi_{T'}(i,l)$. Then, by Lemma 2.(b), $\varphi_T(j,l) = \varphi_{T'}(j,l) - 1$, and then

$$\begin{array}{l} \varphi_T(i,l) = \varphi_{T'}(i,l) = \varphi_{T'}(i,j) = \varphi_T(i,j) - 1 \\ \varphi_T(j,l) = \varphi_{T'}(j,l) - 1 = \varphi_{T'}(i,j) = \varphi_T(i,j) - 1 \\ \varphi_T(k,l) = \varphi_T(i,j) - 1 \\ \delta_T(i) = \delta_T(j) = \delta_T(k) = \delta_T(l) = \varphi_T(i,j) + 1 \end{array}$$

Therefore, the subtree of T rooted at $[k,l]_T$ contains a subtree of the form described in Fig. 3, for at least one leaf h. Moreover, $h \neq k$ because $\varphi_T(h,l) > \varphi_T(j,l) = \varphi_T(k,l)$. But then, again,

$$\varphi_{T'}(l,h) = \varphi_T(l,h) = \varphi_T(i,j) = \varphi_{T'}(i,j) + 1 = \varphi_{T'}(l,j)$$

which is again impossible by the same reason as in (1). Therefore, $\varphi_T(i,l) \neq \varphi_{T'}(i,l)$ and, by symmetry, $\varphi_T(j,k) \neq \varphi_{T'}(j,k)$.

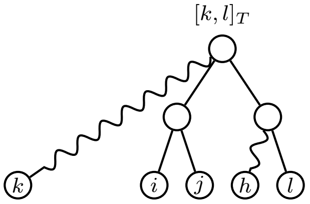

Figure 3: A subtree of the subtree of T rooted at $[k,l]_T$ in case (2) in the proof of Lemma 4.

So,

$$\varphi_T(i,k) \neq \varphi_{T'}(i,k), \varphi_T(i,l) \neq \varphi_{T'}(i,l), \varphi_T(j,k) \neq \varphi_{T'}(j,k), \varphi_T(j,l) \neq \varphi_{T'}(j,l)$$

and thus $D_0(T,T') \geqslant 5$. ☐

Summarizing the last lemmas, we have proved so far that if $D_0(T,T') \leqslant 3$ and $\varphi_T(i,j) \neq \varphi_{T'}(i,j)$, then, up to interchanging T and T', $\varphi_T(i,j) = \varphi_{T'}(i,j) + 1$ and either i and j are sibling in T' or one of these leaves is a sibling of the parent of the other one in T'. Next two lemmas cover these two remaining cases.

Lemma 5. *Let $T, T' \in \mathcal{T}_n$ be such that $D_0(T,T') \leqslant 3$, and assume that $\varphi_T(i,j) = \varphi_{T'}(i,j) + 1$, for some $1 \leqslant i < j \leqslant n$. If i and j are sibling in T', then they are also sibling in T, they have no other sibling in T, and T' is obtained from T by contracting the arc ending in $[i,j]_T$. And then, $D_0(T,T') = 3$.*

Proof. If $\delta_{T'}(i) = \delta_{T'}(j) = \varphi_{T'}(i,j) + 1$, then it must happen that $\delta_T(i) = \delta_{T'}(i) + 1$ and $\delta_T(j) = \delta_{T'}(j) + 1$. Indeed, if $\delta_T(i) \leqslant \delta_{T'}(i)$, then $\delta_T(i) \leqslant \varphi_{T'}(i,j) + 1 = \varphi_T(i,j)$, which is impossible. Therefore, $\delta_T(i) > \delta_{T'}(i)$

and by symmetry $\delta_T(j) > \delta_{T'}(j)$. Since $\varphi_T(i,j) = \varphi_{T'}(i,j) + 1$, $D_0(T,T') \leqslant 3$ implies that $\varphi_T(x,y) = \varphi_{T'}(x,y)$, for every $(x,y) \neq (i,j),(i,i),(j,j)$. Now, if, say $\delta_T(i) \geqslant \delta_{T'}(i) + 2$, then

$$\delta_T(i) \geqslant \delta_{T'}(i) + 2 = \varphi_{T'}(i,j) + 3 = \varphi_T(i,j) + 2$$

and there would exist some leaf k such that $[i,k]_T$ is a child of $[i,j]_T$. But then

$$\varphi_{T'}(i,k) = \varphi_T(i,k) = \varphi_T(i,j) + 1 = \varphi_{T'}(i,j) + 2 = \delta_{T'}(i) + 1,$$

which is impossible. This proves that $\delta_T(i) = \delta_{T'}(i) + 1$ and, by symmetry, $\delta_T(j) = \delta_{T'}(j) + 1$.

So, in summary, $\varphi_T(i,j) = \varphi_{T'}(i,j) + 1$, $\delta_T(i) = \delta_{T'}(i) + 1$, $\delta_T(j) = \delta_{T'}(j) + 1$ and $\varphi_T(x,y) = \varphi_{T'}(x,y)$, for every $(x,y) \neq (i,j),(i,i),(j,j)$, and in particular $d_{\varphi,p}(T,T') = 3$.

Now, $\delta_T(i) = \delta_{T'}(i) + 1 = \varphi_{T'}(i,j) + 2 = \varphi_T(i,j) + 1$, and by symmetry, $\delta_T(j) = \varphi_T(i,j) + 1$, either. Therefore, i and j are sibling in T. Let us see that they have no other sibling in this tree. Indeed, if k is a sibling of i and j in T, then

$$\varphi_{T'}(i,k) = \varphi_T(i,k) = \varphi_T(i,j) = \varphi_{T'}(i,j) + 1 = \delta_{T'}(i)$$

which is impossible.

Let x be the parent of $[i,j]_T$, and assume that the subtree T_0 of T rooted at x is as described in Fig. 4.(a), for some (possibly empty) subtree $\widehat{T}$. Moreover, let T_0' be the subtree of T' rooted at $[i,j]_{T'}$, which is as described in Fig. 4.(b) for some subtree $\widehat{T'}$. We shall prove that $\widehat{T} = \widehat{T'}$.

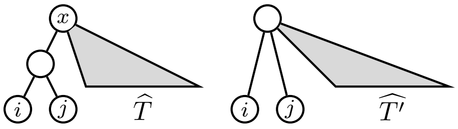

(a) T_0 (b) T_0'

Figure 4: (a) The subtree T_0 of T rooted at the parent of $[i,j]_T$ in the proof of Lemma 5. (b) The subtree T_0' of T' rooted at $[i,j]_{T'}$ in the proof of the same Lemma.

For every $k \in L(\widehat{T})$,

$$\varphi_{T'}(i,k) = \varphi_T(i,k) = \varphi_T(i,j) - 1 = \varphi_{T'}(i,j),$$

which entails that $k \in L(\widehat{T'})$. Conversely, if $k \in L(\widehat{T'})$, then

$$\varphi_T(i,k) = \varphi_{T'}(i,k) = \varphi_{T'}(i,j) = \varphi_T(i,j) - 1,$$

which entails that $k \in L(\widehat{T})$. Thus, $L(\widehat{T}) = L(\widehat{T'})$. And finally, for every (not necessarily different) $k, l \in L(\widehat{T})$,

$$\varphi_{\widehat{T}}(k,l) = \varphi_T(k,l) - \delta_T(x) = \varphi_T(k,l) - \varphi_T(i,j) + 1 = \varphi_{T'}(k,l) - \varphi_{T'}(i,j) = \varphi_{\widehat{T'}}(k,l),$$

which implies by Theorem 1 that $\widehat{T} = \widehat{T'}$ (notice that $\widehat{T}$ and $\widehat{T'}$ can have elementary roots).

Finally, let us prove now that T and T' are exactly the same except for T_0 and T_0'. More specifically, let T_1 and T_1' be obtained by replacing in T and T' the subtrees T_0 and T_0' by a single leaf x. Since for every $p, q \notin L(T_0) = L(T_0')$,

$$\begin{array}{l} \varphi_{T_1'}(p,q) = \varphi_{T'}(p,q) = \varphi_T(p,q) = \varphi_{T_1}(p,q), \\ \varphi_{T_1'}(x,p) = \varphi_{T'}(i,p) = \varphi_T(i,p) = \varphi_{T_1}(p,x), \end{array}$$

we deduce, again by Theorem 1, that $T_1 = T_1'$.

This completes the proof that T' is obtained from T by replacing in it the subtree T_0 rooted at the parent x of $[i,j]_T$ by the subtree T_0' obtained from T_0 by contracting the arc $(x, [i,j]_T)$. □

Lemma 6. *Let $T, T' \in \mathcal{T}_n$ be such that $D_0(T,T') \leqslant 3$. Assume that $\varphi_T(i,j) = \varphi_{T'}(i,j) + 1$, for some $1 \leqslant i < j \leqslant n$, and that j is a sibling of the parent of i in T'. Then, the subtree of T' rooted at $[i,j]_{T'}$ is the tree T_0' depicted in Fig. 5.(a), for some taxon $k \neq i, j$ and some (possibly empty) subtree $\widehat{T'}$, and T is obtained from T' by replacing T_0' by the tree T_0 depicted in Fig. 5.(b). And then, $D_0(T,T') = 3$.*

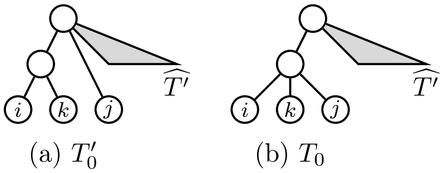

Figure 5: (a) The subtree T_0' of T' rooted at $[i,j]_{T'}$ in the statement of Lemma 6. (b) The subtree T_0 which replaces T_0' in T in the same statement.

Proof. We assume that $\delta_{T'}(i) = \varphi_{T'}(i,j) + 2$ and $\delta_{T'}(j) = \varphi_{T'}(i,j) + 1$. This implies that there exists at least one leaf k such that $[i,k]_{T'} \prec [i,j]_{T'}$. Since $\varphi_T(i,j) = \varphi_{T'}(i,j) + 1$, $|\varphi_T(i,k) - \varphi_{T'}(i,k)| + |\varphi_T(j,k) - \varphi_{T'}(j,k)| \geqslant 1$ and $\delta_T(j) > \delta_{T'}(j)$ (because, otherwise, $\delta_T(j) \leqslant \delta_{T'}(j) = \varphi_{T'}(i,j) + 1 = \varphi_T(i,j)$, which is impossible), $D_0(T,T') \leqslant 3$ entails that $\varphi_T(i,k) = \varphi_{T'}(i,k)$ or $\varphi_T(j,k) = \varphi_T(j,k)$, and that $\varphi_T(x,y) = \varphi_{T'}(x,y)$ for every $(x,y) \neq (i,j), (i,k), (j,k), (j,j)$ (and, in particular, k is the only leaf different from i such that $[i,k]_{T'} \prec [i,j]_{T'}$). Moreover, we have that $D_0(T,T') = 3$.

Let us see now that $\delta_T(j) = \delta_{T'}(j) + 1$. Indeed, if $\delta_T(j) \geqslant \delta_{T'}(j) + 2$, then

$$\delta_T(j) \geqslant \delta_{T'}(j) + 2 = \varphi_{T'}(i,j) + 3 = \varphi_T(i,j) + 2$$

and there would exist some leaf l such that $[j,l]_T$ is a child of $[i,j]_T$. But then

$$\varphi_{T'}(j,l) = \varphi_T(j,l) = \varphi_T(i,j) + 1 = \varphi_{T'}(i,j) + 2 = \delta_{T'}(j) + 1$$

and we reach a contradiction.

So, in summary, the subtree T'_0 of T' rooted a $[i,j]_{T'}$ is as described in Fig. 5.(a), and $\varphi_T(i,j) = \varphi_{T'}(i,j) + 1$, $\delta_T(j) = \delta_{T'}(j) + 1$, $\varphi_T(x,y) = \varphi_{T'}(x,y)$ for every $(x,y) \neq (i,j),(i,k),(j,k),(j,j)$, and either $\varphi_T(i,k) = \varphi_{T'}(i,k)$ or $\varphi_T(j,k) = \varphi_T(j,k)$. Now, we discuss these two possibilities.

(a) If $\varphi_T(j,k) = \varphi_{T'}(j,k)$, then $\varphi_T(i,k) = \varphi_{T'}(i,k) - 1$ by Lemma 2.(b). In this case

$$\begin{array}{l} \varphi_T(i,k) = \varphi_{T'}(i,k) - 1 = \varphi_{T'}(i,j) = \varphi_T(i,j) - 1 \\ \varphi_T(j,k) = \varphi_{T'}(j,k) = \varphi_{T'}(i,j) = \varphi_T(i,j) - 1 \\ \delta_T(i) = \delta_{T'}(i) = \varphi_{T'}(i,j) + 2 = \varphi_T(i,j) + 1 \\ \delta_T(j) = \delta_{T'}(j) + 1 = \varphi_{T'}(i,j) + 2 = \varphi_T(i,j) + 1 \\ \delta_T(k) = \delta_{T'}(k) = \varphi_{T'}(i,j) + 2 = \varphi_T(i,j) + 1 \end{array}$$

This means that the subtree of T rooted at $[i,k]_T = [j,k]_T$ contains a subtree of the form described in Fig. 6, for at least some new leaf h. But then

$$\varphi_{T'}(k,h) = \varphi_T(k,h) = \varphi_T(i,j) = \varphi_{T'}(i,j) + 1 = \varphi_{T'}(i,k)$$

which is impossible in T', because i and k are the only descendants of $[i,k]_{T'}$ in T'. So, this case is impossible.

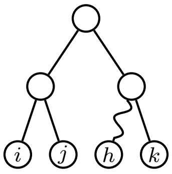

Figure 6: A subtree contained in the subtree of T rooted at $[i,j]_T$ in case (a) in the proof of Lemma 6.

(b) If $\varphi_T(i,k) = \varphi_{T'}(i,k)$, then $\varphi_T(j,k) = \varphi_{T'}(j,k) + 1$ Lemmas 2.(a) and 3. In this case

$$\begin{array}{l} \varphi_T(i,k) = \varphi_{T'}(i,k) = \varphi_{T'}(i,j) + 1 = \varphi_T(i,j) \\ \varphi_T(j,k) = \varphi_{T'}(j,k) + 1 = \varphi_{T'}(i,j) + 1 = \varphi_T(i,j) \\ \delta_T(i) = \delta_T(j) = \delta_T(k) = \varphi_T(i,j) + 1 \text{ as in (a)} \end{array}$$

This implies that i, j, k are sibling in T. If l is any other sibling of them in T, then

$$\varphi_{T'}(i,l) = \varphi_T(i,l) = \varphi_T(i,k) = \varphi_{T'}(i,k)$$

which entails that l is another descendant of $[i,k]_{T'}$ in T', which is impossible. Therefore, the subtree T_0 of T rooted at the parent of $[i,j]_T$ has the form depicted in Fig. 7, for some subtree $\widehat{T}$.

Finally, the same argument as in the last part of the proof of the last lemma shows that $\widehat{T} = \widehat{T'}$, and that if T_1 and T_1' are obtained by replacing in T and T' the subtrees T_0 and T_0' by a single leaf x, then $T_1 = T_1'$. We leave the details to the reader.

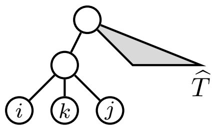

Figure 7: The subtree T_0 rooted at the parent of $[i,j]_T$ in case (b) in the proof of Lemma 6.

This completes the proof that T and T' are as described in the statement. $\square$

We have proved so far that the minimum value of D_0 on $\mathcal{T}_n$ is 3, and we have characterized those pairs of trees $T, T' \in \mathcal{T}_n$ such that $D_0(T,T') = 3$. To extend this result to every D_p, $p \geqslant 1$, it is enough to check that every pair of trees in $\mathcal{T}_n$ such that $D_0(T,T') = 3$ also satisfies that $D_p(T,T') = 3$ for every $p \geqslant 1$, which is straightforward. This completes the proof of Proposition 2.

Proof of Proposition 3

As in Proposition 2, we also split this proof into several lemmas. First of all, notice that there are pairs of trees $T, T' \in \mathcal{BT}_n$ such that $D_p(T,T') = 4$ for every $p \in \{0\} \cup [1, \infty[$: see, for instance, Fig. 8. Therefore, the minimum value of D_p on $\mathcal{BT}_n$ is at most 4.

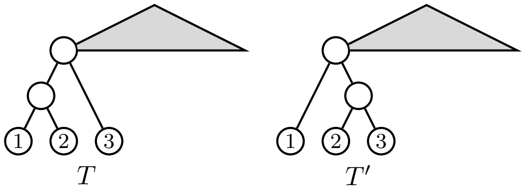

Figure 8: A pair of binary trees such that $D_p(T,T') = 4$. The grey triangles represent the same tree.

Notice also that Lemma 1 also applies in $\mathcal{BT}_n$, and therefore, if $T, T' \in \mathcal{BT}_n$ are such that $D_0(T, T') > 0$, then there exist two taxa $i \neq j$ such that $\varphi_T(i,j) \neq \varphi_{T'}(i,j)$. And, of course, Lemma 2 also applies in $\mathcal{BT}_n$.

Lemma 7. *Let $T, T' \in \mathcal{BT}_n$ be such that $D_0(T, T') \leqslant 4$. If $\varphi_T(i,j) = \varphi_{T'}(i,j) + m$, for some $1 \leqslant i < j \leqslant n$ and some $m \geqslant 1$, then $m = 1$.*

Proof. Assume that $\varphi_T(i,j) = \varphi_{T'}(i,j) + m$ with $m \geqslant 2$, and let us reach a contradiction.

If $\delta_{T'}(i) = \delta_T(i)$, then $\delta_{T'}(i) > \varphi_T(i,j) = \varphi_{T'}(i,j) + m$, and therefore there exist leaves $x_1, \ldots, x_m$ such that $\varphi_T(i, x_l) = \varphi_{T'}(i,j) + l$, for $l = 1, \ldots, m$. By Lemma 2, each such leaf x_l adds at least 1 to $D_0(T, T')$. Therefore $D_0(T, T') \geqslant 1 + m$. Now, if moreover $\delta_{T'}(j) = \delta_T(j)$, then there also exist leaves $y_1, \ldots, y_m$ such that $\varphi_T(j, y_l) = \varphi_{T'}(i,j) + l$, for $l = 1, \ldots, m$, and each such leaf y_l also adds at least 1 to $D_0(T, T')$, which entails $D_0(T, T') \geqslant 1 + 2m \geqslant 5$. So, if $D_0(T, T') \leqslant 4$, it must happen that $\delta_{T'}(i) \neq \delta_T(i)$ or $\delta_{T'}(j) \neq \delta_T(j)$ (or both). Let assume that $\delta_{T'}(j) \neq \delta_T(j)$.

Now, $\varphi_T(i,j) = \varphi_{T'}(i,j) + m \geqslant m$, and therefore there exist leaves $z_1, \ldots, z_m$ such that $\varphi_T(i, z_l) = \varphi_T(j, z_l) = \varphi_T(i,j) - l$, for $l = 1, \ldots, m$. If $\varphi_T(i, k_l) = \varphi_{T'}(i, k_l)$, then

$$\varphi_{T'}(i, k_l) = \varphi_T(i, k_l) = \varphi_T(i,j) - l = \varphi_{T'}(i,j) + (m - l) \geqslant \varphi_{T'}(i,j)$$

and therefore, by Lemma 2, $\varphi_{T'}(j, k_l) \neq \varphi_T(j, k_l)$, and thus, each such leaf z_l adds at least 1 to $D_0(T, T')$, which entails $D_0(T, T') \geqslant 2 + m$. Therefore, if $D_0(T, T') \leqslant 4$ and $m \geqslant 2$, it must happen $m = 2$ and, moreover, $\varphi_T(a,b) = \varphi_{T'}(a,b)$ for every $(a,b) \neq (i,j), (j,j), (i,z_1), (i,z_2), (j,z_1), (j,z_2)$.

In particular, $\delta_T(i) = \delta_{T'}(i)$, which as we have seen implies that there are at least two leaves x_1, x_2 such that $i \prec [i, x_2]_{T'} \prec [i, x_1]_{T'} \prec [i,j]_{T'}$. Since

$$\varphi_{T'}(z_1, z_2) = \varphi_T(z_1, z_2) = \varphi_T(i,j) - 2 = \varphi_{T'}(i,j)$$

implies that (up to interchanging z_1 and z_2) $i \prec [i, z_1]_{T'} \prec [i,j]_{T'}$ and $j \prec [j, z_2]_{T'} \prec [i,j]_{T'}$, we conclude that $\{x_1, x_2, z_1, z_2\}$ are at least 3 different leaves and hence they contribute at least 3 to $D_0(T, T')$, making $D_0(T, T') \geqslant 5$. □

Lemma 8. *Let $T, T' \in \mathcal{BT}_n$ be such that $D_0(T, T') \leqslant 4$. If $\varphi_T(i,j) = \varphi_{T'}(i,j) + 1$, for some $1 \leqslant i < j \leqslant n$, then $\delta_{T'}(i), \delta_{T'}(j) \leqslant \varphi_{T'}(i,j) + 2$.*

Proof. Let us assume that $\delta_{T'}(i) \geqslant \varphi_{T'}(i,j) + 3$, and let us reach a contradiction. The case when $\delta_{T'}(j) \geqslant \varphi_{T'}(i,j) + 3$ is symmetrical.

Since $\varphi_T(i,j) = \varphi_{T'}(i,j) + 1 > 0$, there exists some taxon k_0 such that $[i,k_0]_T$ is the parent of $[i,j]_T$. Let us distinguish several cases.

(a) Assume that $\varphi_T(i,k_0) = \varphi_{T'}(i,k_0)$. Then, $\varphi_{T'}(i,k_0) = \varphi_T(i,k_0) = \varphi_T(i,j) - 1 = \varphi_{T'}(i,j)$ implies that $[j,k_0]_{T'} \prec [i,j]_{T'}$ and thus $\varphi_{T'}(j,k_0) > \varphi_{T'}(i,j) = \varphi_T(i,j) - 1 = \varphi_T(j,k_0)$ and in particular, by the previous lemma $\varphi_{T'}(j,k_0) = \varphi_T(j,k_0) + 1 = \varphi_T(i,j) = \varphi_{T'}(i,j) + 1$. Now, since $D_0(T,T') \leqslant 4$, by Lemma 3 the number of leaves $a \neq i,j,k_0$ such that $a \prec [i,j]_{T'}$ is at most 2.

If $\delta_{T'}(i) \geqslant \varphi_{T'}(i,j) + 3$, then there exist leaves k_1, k_2 such that $\varphi_{T'}(i,k_1) = \varphi_{T'}(i,j) - 1$ and $\varphi_{T'}(i,k_2) = \varphi_{T'}(i,j) - 2$ and then $\varphi_T(x,y) = \varphi_{T'}(x,y)$ for every $(x,y) \neq (i,j),(i,k_0),(j,k_0),(k_1,i),(k_1,j),(k_2,i),(k_2,j)$. In particular, no leaf other than i,j,k_0,k_1,k_2 descends from $[i,j]_{T'}$. But then

$$\begin{array}{l} \varphi_T(k_1,k_0) = \varphi_{T'}(k_1,k_0) = \varphi_{T'}(i,j) = \varphi_T(i,j) - 1, \quad \varphi_T(k_2,k_0) = \varphi_T(i,j) - 1 \\ \varphi_T(k_1,k_2) = \varphi_{T'}(k_1,k_2) = \varphi_{T'}(i,j) + 1 = \varphi_T(i,j) \end{array}$$

imply that, up to interchanging k_1 and k_2, $i \prec [i,k_1]_T \prec [i,j]_T$ and $j \prec [j,k_2]_T \prec [i,j]_T$, and then

$$\delta_{T'}(j) = \delta_T(j) > \varphi_T(i,j) + 1 = \varphi_{T'}(i,j) + 2$$

implies the existence of at least another leaf h such that $j \prec [j,h]_{T'} \prec [j,k_0]_{T'} \prec [i,j]_{T'}$, which, as we have mentioned, is impossible. So, this case cannot happen.

(b) Assume now that $\varphi_T(j,k_0) = \varphi_{T'}(j,k_0)$. By symmetry with the previous case, this implies that $\varphi_{T'}(i,k_0) = \varphi_{T'}(i,j) + 1$, $\varphi_{T'}(i,k_0) = \varphi_T(i,k_0) + 1$ and that the number of leaves $a \neq i,j,k_0$ such that $a \prec [i,j]_{T'}$ is at most 2. Now we have three new subcases to discuss.

(b.1) If $\delta_{T'}(i) = \varphi_{T'}(i,j) + 4$, so that there exist leaves $k_1, k_2 \neq i$ such that $\varphi_{T'}(i,k_0), \varphi_{T'}(i,k_1), \varphi_{T'}(i,k_2) > \varphi_{T'}(i,j)$, and no leaf other that i,j,k_0,k_1,k_2 descends from $[i,j]_{T'}$. Then $\varphi_T(x,y) = \varphi_{T'}(x,y)$ for every $(x,y) \neq (i,j),(i,k_0),(j,k_0),(k_1,i),(k_1,j),(k_2,i),(k_2,j)$. But in this case it must happen that $\delta_T(j) = \delta_{T'}(j) = \varphi_{T'}(i,j) + 1 = \varphi_T(i,j)$, which is impossible. So, this case cannot happen.

(b.2) If $\delta_{T'}(i) = \varphi_{T'}(i,j) + 3$ and $\delta_{T'}(j) = \varphi_{T'}(i,j) + 2$, so that there exist leaves k_1, k_2 such that $\varphi_{T'}(j,k_1) = \varphi_{T'}(i,j) + 1$, $\varphi_{T'}(i,k_2) = \varphi_{T'}(i,j) + 2$ and, recall, $\varphi_{T'}(i,k_0) = \varphi_{T'}(i,j) + 1$, then $\varphi_T(x,y) = \varphi_{T'}(x,y)$ for every $(x,y) \neq (i,j),(i,k_0),(j,k_0),(k_1,i),(k_1,j),(k_2,i),(k_2,j)$. But then

$$\varphi_T(k_1,k_0) = \varphi_{T'}(k_1,k_0) = \varphi_{T'}(i,j) = \varphi_T(i,j) - 1$$

implies that $k_1 \prec [i,j]_T$, and then

$$\delta_T(j) = \delta_{T'}(j) = \varphi_{T'}(i,j) + 2 = \varphi_T(i,j) + 1,$$
$$\delta_T(k_1) = \delta_{T'}(k_1) = \varphi_{T'}(i,j) + 2 = \varphi_T(i,j) + 1$$

imply that j and k_1 are the only children of $[i,j]_T$, which is, of course, impossible. So, this case cannot happen, either.

(b.3) If $\delta_{T'}(i) = \varphi_{T'}(i,j)+3$ and $\delta_{T'}(j) = \varphi_{T'}(i,j)+1$, then on the one hand there exists a leaf k_1 such that $\varphi_{T'}(i,k_1) = \varphi_{T'}(j,k_0) - 1 = \varphi_{T'}(i,j) - 2$ and, on the other hand, as we have seen in (b.1), $\delta_T(j) > \delta_{T'}(j)$. Then, $\varphi_T(x,y) = \varphi_{T'}(x,y)$ for every $(x,y) \neq (i,j),(j,j),(i,k_0),(j,k_0),(k_1,i),(k_1,j)$, and in particular no leaf other than i,j,k_0,k_1 descends from $[i,j]_{T'}$.

Now,

$$\varphi_T(k_1,k_0) = \varphi_{T'}(k_1,k_0) = \varphi_{T'}(i,j) + 1 = \varphi_T(i,j)$$

implies that $k_1 \not\prec [i,j]_T$, and

$$\delta_T(i) = \delta_{T'}(i) = \varphi_{T'}(i,j) + 3 = \varphi_T(i,j) + 2$$

implies that there exists a leaf $h \neq k_0, k_1$ such that $i \prec [i,h]_T \prec [i,j]_T$ and hence

$$\varphi_{T'}(i,h) = \varphi_T(i,h) > \varphi_T(i,j) + 1 = \varphi_{T'}(i,j)$$

would entail that $h \prec [i,j]_{T'}$, which is impossible. Thus, this case cannot happen, either.

(c) Assume finally that $\varphi_T(i,k_0) \neq \varphi_{T'}(i,k_0)$ and $\varphi_T(j,k_0) \neq \varphi_{T'}(j,k_0)$. The contribution to D_0 of the pairs $(i,j),(i,k_0),(j,k_0)$ is at least 3, and therefore there can only exist at most one other pair of leaves with different cophenetic value in T and in T'. Since every $x \neq i,j$ such that $x \prec [i,j]_{T'}$ defines at least one such pair, we conclude that if $\delta_{T'}(i) \geqslant \varphi_{T'}(i,j) + 3$, then, it must happen that $[i,k_0]_{T'} \prec [i,j]_{T'}$ and that there can only exist one leaf $k_1 \neq k_0, i$ such that $[i,k_1]_{T'} \prec [i,j]_{T'}$, and then, moreover $[i,k_0]_{T'} \neq [i,k_1]_{T'}$. In this case, $\varphi_T(x,y) = \varphi_{T'}(x,y)$ for every $(x,y) \neq (i,j),(i,k_0),(j,k_0),(k_1,i),(k_1,j)$. But then, in particular, $\delta_{T'}(j) = \varphi_{T'}(i,j) + 1$ and $\delta_T(j) = \delta_{T'}(j)$, which implies $\delta_T(i) = \varphi_T(i,j)$, which is impossible

This finishes the proof that, if $D_0(T,T') \leqslant 4$, then $\delta_{T'}(i) \leqslant \varphi_{T'}(i,j) + 2$ and $\delta_{T'}(j) \leqslant \varphi_{T'}(i,j) + 2$. □

Lemma 9. *Let $T, T' \in \mathcal{BT}_n$ be such that $D_0(T,T') \leqslant 4$. If $\varphi_T(i,j) = \varphi_{T'}(i,j) + 1$, for some $1 \leqslant i < j \leqslant n$, then i,j are sibling in T.*

Proof. Let k_0 be any leaf such that $[i,k_0]_T = [j,k_0]_T$ is the parent of $[i,j]_T$ in T. If $\varphi_T(i,k_0) = \varphi_{T'}(i,k_0)$, then $\varphi_{T'}(i,k_0) = \varphi_T(i,k_0) = \varphi_T(i,j) - 1 = \varphi_{T'}(i,j)$ implies that $[j,k_0]_{T'} \prec [i,j]_{T'}$ and thus $\varphi_{T'}(j,k_0) > \varphi_{T'}(i,j) = \varphi_T(i,j) - 1 = \varphi_T(j,k_0)$. Therefore, $|\varphi_T(i,k_0) - \varphi_{T'}(i,k_0)| + |\varphi_T(j,k_0) - \varphi_{T'}(j,k_0)| \geqslant 1$.

Assume now that i,j are not sibling in T, and let h be a leaf such that $[i,h]_T$ is a child of $[i,j]_T$. If $\varphi_T(i,h) \leqslant \varphi_{T'}(i,h)$, then

$$\delta_{T'}(i) \geqslant \varphi_{T'}(i,h) + 1 \geqslant \varphi_T(i,h) + 1 = \varphi_T(i,j) + 2 = \varphi_{T'}(i,j) + 3$$

which is impossible by the previous lemma. Therefore, $\varphi_T(i,h) > \varphi_{T'}(i,h)$, and by Lemma 7, $\varphi_T(i,h) = \varphi_{T'}(i,h) + 1$.

In a similar way, if $\delta_T(i) = \delta_{T'}(i)$, then

$$\delta_{T'}(i) = \delta_T(i) \geqslant \varphi_T(i,h) + 1 = \varphi_T(i,j) + 2 = \varphi_{T'}(i,j) + 3$$

which is again impossible by the previous lemma. Therefore, $\delta_T(i) \neq \delta_{T'}(i)$, too. So, (i,j), (i,k_0), (j,k_0), (i,i), and (i,h) contribute at least 4 to $D_0(T,T') \leqslant 4$, which implies that $\varphi_T(x,y) = \varphi_{T'}(x,y)$ for every other pair of leaves (x,y). But then,

$$\begin{aligned}\varphi_{T'}(j,h) = \varphi_T(j,h) = \varphi_T(i,j) = \varphi_{T'}(i,j) + 1\\ \varphi_{T'}(i,h) = \varphi_T(i,h) - 1 = \varphi_T(i,j) = \varphi_{T'}(i,j) + 1\end{aligned}$$

which is impossible. Therefore, i and j are sibling in T. □

Lemma 10. *Let $T, T' \in \mathcal{BT}_n$ be such that $D_0(T,T') \leqslant 4$. If $\varphi_T(i,j) = \varphi_{T'}(i,j)+1$, for some $1 \leqslant i < j \leqslant n$, then i,j are not sibling in T'.*

Proof. Assume that i,j are sibling in T', and recall that we already know that they are sibling in T. Let k_0 be any leaf such that $[i,k_0]_T = [j,k_0]_T$ is the parent of $[i,j]_T$ in T. If $\varphi_T(i,k_0) = \varphi_{T'}(i,k_0)$, then

$$\varphi_{T'}(i,k_0) = \varphi_T(i,k_0) = \varphi_T(i,j) - 1 = \varphi_{T'}(i,j)$$

which is impossible if i,j are sibling in T'. Thus, $\varphi_T(i,k_0) \neq \varphi_{T'}(i,k_0)$ and, by symmetry, $\varphi_T(j,k_0) \neq \varphi_{T'}(j,k_0)$. On the other hand, if $\delta_T(i) = \delta_{T'}(i)$, then

$$\delta_T(i) = \delta_{T'}(i) = \varphi_{T'}(i,j) + 1 = \varphi_T(i,j)$$

which is also impossible. Therefore, $\delta_T(i) \neq \delta_{T'}(i)$ and, by symmetry, $\delta_T(j) \neq \delta_{T'}(j)$. But, then, $D_0(T,T') \geqslant 5$. □

Summarizing what we know so far, we have proved that if $D_0(T,T') \leqslant 4$ and $\varphi_T(i,j) \neq \varphi_{T'}(i,j)$, then, up to interchanging T and T', $\varphi_T(i,j) = \varphi_{T'}(i,j)+1$, i,j are sibling in T, and then the subtree of T' rooted at $[i,j]_{T'}$ is a triplet or a totally balanced quartet; cf. Fig. 9. Next two lemmas cover these two possibilities.

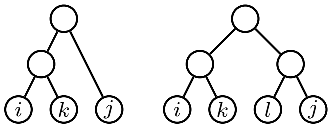

Figure 9: The only possibilities for the subtree of T' rooted at $[i,j]_{T'}$ if $D_0(T,T') \leqslant 4$ and $\varphi_T(i,j) = \varphi_{T'}(i,j)+1$.

Lemma 11. *Let $T,T' \in \mathcal{BT}_n$ be such that $D_0(T,T') \leqslant 4$. If $\varphi_T(i,j) = \varphi_{T'}(i,j)+1$, for some $1 \leqslant i < j \leqslant n$, and the subtree of T' rooted at $[i,j]_{T'}$ is the triplet depicted in the left hand side of Fig. 9, then T is obtained from T' by interchanging j and k: cf. Fig. 10. And, then $D_0(T,T') = 4$.*

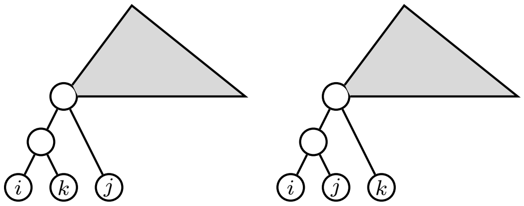

Figure 10: The only pairs of trees T,T' such that $D_0(T,T') \leqslant 4$ and $\varphi_T(i,j) = \varphi_{T'}(i,j)+1$, when the subtree of T' rooted at $[i,j]_{T'}$ is a triplet.

Proof. Assume that the subtree of T' rooted at $[i,j]_{T'}$ has the form depicted in the left hand side of Fig. 9, and that $\varphi_T(i,j) = \varphi_{T'}(i,j)+1$. Then, since i and j are sibling in T,

$$\delta_T(j) = \varphi_T(i,j)+1 = \varphi_{T'}(i,j)+2 = \delta_{T'}(j)+1.$$

Now, if $\varphi_T(i,k) \geqslant \varphi_{T'}(i,k)$, then

$$\varphi_T(i,k) \geqslant \varphi_{T'}(i,k) = \varphi_{T'}(i,j)+1 = \varphi_T(i,j)$$

which is impossible, because i and j are sibling in T. Therefore, $\varphi_T(i,k) < \varphi_{T'}(i,k)$ and, by Lemma 7, $\varphi_T(i,k) = \varphi_{T'}(i,k)-1$, and in particular $\varphi_T(i,k) = \varphi_T(j,k) = \varphi_T(i,j)-1$. Therefore, $[i,k]_T$ is the parent of $[i,j]_T$ in T.

Finally, if $\delta_T(k) \geqslant \varphi_T(i,j)+1$, then there exists at least some other leaf $l \prec [i,k]_T = [j,k]_T$. But then $\varphi_T(i,l) \neq \varphi_{T'}(i,l)$, because otherwise

$$\varphi_{T'}(i,l) = \varphi_T(i,l) = \varphi_T(i,j) - 1 = \varphi_{T'}(i,j),$$

which is impossible because the only leaves descending from $[i,j]_{T'}$ are i,j,k. And, by symmetry $\varphi_T(j,l) \neq \varphi_{T'}(j,l)$, and we reach $D_0(T,T') \geqslant 5$. Therefore,

$$\delta_T(k) = \varphi_T(i,j) = \varphi_{T'}(i,j) + 1 = \delta_{T'}(k) - 1.$$

So, in summary, $\varphi_T(i,j) = \varphi_{T'}(i,j)+1$, $\delta_T(j) = \delta_{T'}(j)+1$, $\varphi_T(i,k) = \varphi_{T'}(i,k)-1$, and $\delta_T(k) = \delta_{T'}(k)-1$, and $\varphi_T(x,y) = \varphi_{T'}(x,y)$ for every (x,y) other than $(i,j),(j,j),(i,k),(k,k)$. Moreover, in T, k is the other child of the parent of $[i,j]_T$.

So, the subtree T_0 of T rooted at the parent of $[i,j]_T$ is obtained by interchanging j and k in the subtree T_0' of T' rooted at $[i,j]_{T'}$. Finally, let us prove now that T and T' are exactly the same except for T_0 and T_0'. More specifically, let T_1 and T_1' be obtained by replacing in T and T' the subtrees T_0 and T_0' by a single leaf x. Since for every $p,q \notin \{i,j,k\}$,

$$\begin{aligned}\varphi_{T_1'}(p,q) = \varphi_{T'}(p,q) = \varphi_T(p,q) = \varphi_{T_1}(p,q),\\ \varphi_{T_1'}(x,p) = \varphi_{T'}(i,p) = \varphi_T(i,p) = \varphi_{T_1}(x,p),\end{aligned}$$

we deduce, by Theorem 1, that $T_1 = T_1'$.

This completes the proof that T is obtained from T' by interchanging the leaf j and its nephew k. $\square$

Lemma 12. *Let $T,T' \in \mathcal{BT}_n$ be such that $D_0(T,T') \leqslant 4$. If $\varphi_T(i,j) = \varphi_{T'}(i,j)+1$, for some $1 \leqslant i < j \leqslant n$, and the subtree of T' rooted at $[i,j]_{T'}$ is the quartet depicted in the right hand side of Fig. 9, then T is obtained from T' by interchanging j and k: cf. Fig. 11. And, then $D_0(T,T') = 4$.*

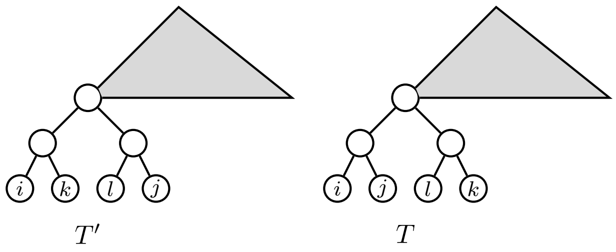

Figure 11: The only pairs of trees T,T' such that $D_0(T,T') \leqslant 4$ and $\varphi_T(i,j) = \varphi_{T'}(i,j)+1$, when the subtree of T' rooted at $[i,j]_{T'}$ is a quartet.

Proof. Assume that the subtree of T' rooted at $[i,j]_{T'}$ has the form depicted in the right hand side of Fig. 9, and that $\varphi_T(i,j) = \varphi_{T'}(i,j) + 1$.

If $\varphi_T(i,k) \geqslant \varphi_{T'}(i,k)$, then

$$\varphi_T(i,k) \geqslant \varphi_{T'}(i,k) = \varphi_{T'}(i,j) + 1 = \varphi_T(i,j)$$

which is impossible if i,j are sibling in T. Therefore, $\varphi_T(i,k) < \varphi_{T'}(i,k)$ and, by Lemma 7, $\varphi_T(i,k) = \varphi_{T'}(i,k) - 1$, and in particular $\varphi_T(i,k) = \varphi_T(i,j) - 1$. By symmetry, $\varphi_T(j,l) = \varphi_{T'}(j,l) - 1$ and hence $\varphi_T(j,l) = \varphi_T(i,j) - 1$, too. Therefore, both k and l are descendants of the parent of $[i,j]_T$. But then,

$$\varphi_{T'}(k,l) = \varphi_{T'}(i,j) = \varphi_T(i,j) - 1 < \varphi_T(k,l)$$

and therefore, by Lemma 7, $\varphi_T(k,l) = \varphi_{T'}(k,l) + 1 = \varphi_T(i,j)$.

At this point, $D_0(T,T') \leqslant 4$ entails that $\varphi_T(x,y) = \varphi_{T'}(x,y)$ for every (x,y) other than $(i,j),(i,k),(j,l),(k,l)$. Moreover, i,k,j,l are the only descendant leaves of the parent of $[i,j]_T$ in T. Indeed, if h is another descendant leaf of the parent of $[i,j]_{T'}$, then

$$\varphi_{T'}(i,h) = \varphi_T(i,h) = \varphi_T(i,j) - 1 = \varphi_{T'}(i,j)$$

and therefore h would be another descendant of $[i,j]_{T'}$. And, as we have seen, the subtree T_0 of T rooted at this node is obtained from the subtree T_0' of T' rooted at $[i,j]_{T'}$ by interchanging j and k. Finally, arguing as in the last part of the proof of the previous lemma, we deduce that T and T' are exactly the same except for T_0 and T_0'. □

We have proved so far that the minimum value of D_0 on $\mathcal{BT}_n$ is 4, and we have characterized the pairs of trees $T,T' \in \mathcal{BT}_n$ such that $D_0(T,T') = 4$. To extend this result to every D_p, $p \geqslant 1$, it is enough to check that every pair of binary trees such that $D_0(T,T') = 4$ also satisfies that $D_p(T,T') = 4$ for every $p \geqslant 1$, which is straightforward. This completes the proof of Proposition 3.

Proof of Proposition 4

Let X_n denote any space $\mathcal{UT}_n$, $\mathcal{T}_n$ or $\mathcal{BT}_n$, and let $\Delta_p(X_n)$, $p \in \{0\} \cup [1,\infty[$, denote the diameter of $d_{\varphi,p}$ on X_n.

We consider first the case $p=1$, which will be used later to prove the case $p>1$. For every $T \in \mathcal{UT}_n$, let

$$S(T) = \sum_{i=1}^{n} \delta_T(i), \qquad \Phi(T) = \sum_{1 \leqslant i < j \leqslant n} \varphi_T(i,j).$$

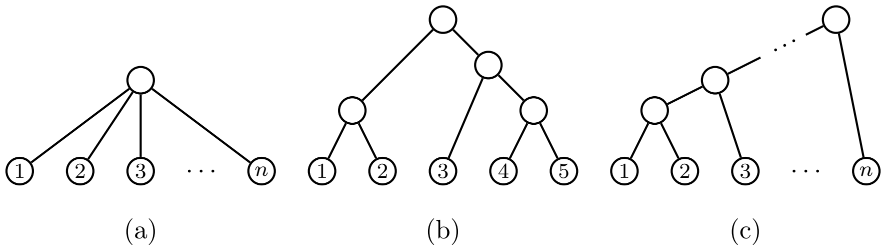

Figure 12: (a) The rooted star with n leaves. (b) The only maximally balanced tree with 5 leaves, up to relabelings. (c) A rooted caterpillar with n leaves.

S and Φ are the extensions to $\mathcal{UT}_n$ of the *Sackin index* [3] and the *total cophenetic index* [1] for phylogenetic trees without nested taxa, respectively. Notice that $\|\varphi(T)\|_1 = S(T) + \Phi(T)$. We have the following results on these indices:

- It is straightforward to check that the minimum values of $S(T)$ and $\Phi(T)$ on $\mathcal{T}_n$ are both reached at the *rooted star tree* with n leaves (the phylogenetic tree with all its leaves of depth 1; see Fig. 12.(a)), and these minimum values are, respectively,

$$\min S(\mathcal{T}_n) = n, \quad \min \Phi(\mathcal{T}_n) = 0.$$

- It is also straightforward to check that the minimum values of $S(T)$ and $\Phi(T)$ on $\mathcal{UT}_n$ are both reached at the rooted star tree with $n-1$ leaves and with the root labeled with n, and these minimum values are, respectively,

$$\min S(\mathcal{UT}_n) = n-1, \quad \min \Phi(\mathcal{UT}_n) = 0.$$

- The minimum values of $S(T)$ and $\Phi(T)$ on $\mathcal{BT}_n$ are both reached at the *maximally balanced trees* with n leaves (those binary trees such that, for every internal node, the numbers of descendant leaves of its two children differ at most in 1; see, for instance, Fig. 12.(b)). And then, these minimum values are, respectively,

$$\min S(\mathcal{BT}_n) = n\lfloor \log_2(4n)\rfloor - 2^{\lfloor \log_2(2n)\rfloor}$$
$$\min \Phi(\mathcal{BT}_n) = \sum_{k=0}^{n-1} a(k), \text{ where } a(k) \text{ is the highest power of 2 that divides } n!$$

For the proofs, see [4] combined with [2] for S, and [1] for Φ. From the first formula it is clear that $\min S(\mathcal{BT}_n)$ is in $\Theta(n\log(n))$. As far as $\min \Phi(\mathcal{BT}_n)$ goes, it is shown in [1] that it satisfies the recurrence

$$\min \Phi(\mathcal{BT}_n) = \min \Phi(\mathcal{BT}_{\lceil n/2\rceil}) + \min \Phi(\mathcal{BT}_{\lfloor n/2\rfloor}) + \binom{\lceil n/2\rceil}{2} + \binom{\lfloor n/2\rfloor}{2}, \quad \text{for } n \geqslant 3$$

from where it is obvious that its order is in $\Theta(n^2)$.

- The maximum values of $S(T)$ and $\Phi(T)$ on both $\mathcal{T}_n$ and $\mathcal{BT}_n$ are reached at the *rooted caterpillar trees* with n leaves (binary phylogenetic trees such that all their internal nodes have a leaf child; see Fig. 12.(c)). And then, these maximum values are, respectively,

$$\max S(\mathcal{T}_n) = \max S(\mathcal{BT}_n) = \binom{n+1}{2} - 1, \quad \max \Phi(\mathcal{T}_n) = \max \Phi(\mathcal{BT}_n) = \binom{n}{3},$$

  which are thus in $\Theta(n^2)$ and $\Theta(n^3)$, respectively. For the proofs, see again [4] for S and [1] for Φ.

- Given any tree in $\mathcal{UT}_n$ with a nested taxon, if we replace this nested taxon by a new leaf labeled with it pending from the node previously labeled with it (cf. Fig. 13), we obtain a new tree in $\mathcal{UT}_n$ with strictly larger value of S and the same value of Φ. This shows that the maximum values of $S(T)$ and $\Phi(T)$ on $\mathcal{UT}_n$ are reached at trees in $\mathcal{T}_n$, and hence at the rooted caterpillar trees with n leaves. Therefore, they are also in $\Theta(n^2)$ and $\Theta(n^3)$, respectively.

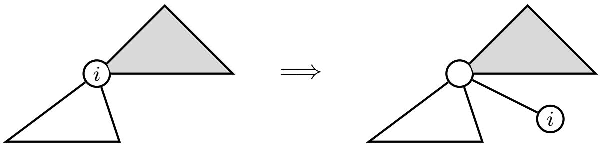

Figure 13: This operation increases the value of S and does not modify the value of Φ.

From these properties we deduce the following result.

Lemma 13. *The minimum value of* $\|\varphi(T)\|_1$ *on* $\mathcal{UT}_n$ *and* $\mathcal{T}_n$ *is in* $\Theta(n)$. *The minimum value of* $\|\varphi(T)\|_1$ *on* $\mathcal{BT}_n$ *is at most in* $\Theta(n^2)$. *The maximum value of* $\|\varphi(T)\|_1$ *on* $\mathcal{UT}_n$, $\mathcal{T}_n$ *and* $\mathcal{BT}_n$ *is in* $\Theta(n^3)$. □

Now, we can apply this lemma to find the order of the diameter of $d_{\varphi,1}$ on the spaces X_n of unweighted phylogenetic trees.

Lemma 14. *The diameter of* $d_{\varphi,1}$ *on* $\mathcal{UT}_n$, $\mathcal{T}_n$ *and* $\mathcal{BT}_n$ *is in* $\Theta(n^3)$.

Proof. Let $T_1, T_2 \in X_n$. Then, on the one hand,

$$d_{\varphi,1}(T_1, T_2) = \|\varphi(T_1) - \varphi(T_2)\|_1 \leqslant \|\varphi(T_1)\|_1 + \|\varphi(T_2)\|_1 \leqslant 2 \cdot \max \|\varphi(X_n)\|_1 = \Theta(n^3)$$

which shows that $\Delta_1(X_n) \leqslant O(n^3)$. On the other hand, if $\|\varphi(T_1)\|_1 \geqslant \|\varphi(T_2)\|_1$, then

$$d_{\varphi,1}(T_1, T_2) = \|\varphi(T_1) - \varphi(T_2)\|_1 \geqslant \|\varphi(T_1)\|_1 - \|\varphi(T_2)\|_1$$

and therefore $\Delta_1(X_n) \geqslant \max \|\varphi(X_n)\|_1 - \min \|\varphi(X_n)\|_1$, which is again in $O(n^3)$. This shows that $\Delta_1(X_n)$ is in $\Theta(n^3)$, as we claimed. □

Let us consider now the case $p > 1$. Since, for every $x \in \mathbb{R}^m$, $\|x\|_1 \leqslant m^{1-\frac{1}{p}} \|x\|_p$, we have that, for every pair of trees $T_1, T_2 \in X_n$,

$$d_{\varphi,1}(T_1, T_2) \leqslant \binom{n+1}{2}^{1-\frac{1}{p}} d_{\varphi,p}(T_1, T_2).$$

and therefore

$$\Delta_1(X_n) \leqslant \binom{n+1}{2}^{1-\frac{1}{p}} \Delta_p(X_n),$$

from where we deduce that

$$\Delta_p(X_n) \geqslant \Delta_1(X_n) \cdot \binom{n+1}{2}^{-1+\frac{1}{p}} = O(n^{(p+2)/p}).$$

To prove the converse inequality, let

$$\varphi^{(p)}(T) = \sum_{1 \leqslant i \leqslant j \leqslant n} \varphi_T(i,j)^p.$$

We have that, for every $T_1, T_2 \in X_n$,

$$\begin{aligned} d_{\varphi,p}(T_1, T_2) &= \|\varphi(T_1) - \varphi(T_2)\|_p \leqslant \|\varphi(T_1)\|_p + \|\varphi(T_2)\|_p = \sqrt[p]{\varphi^{(p)}(T_1)} + \sqrt[p]{\varphi^{(p)}(T_2)} \\ &\leqslant 2\sqrt[p]{\max \varphi^{(p)}(X_n)}, \end{aligned}$$

which implies that $\Delta_p(X_n) \leqslant 2\sqrt[p]{\max \varphi^{(p)}(X_n)}$. Therefore, to prove that the diameter of $d_{\varphi,p}$ on each X_n is bounded from above by $O(n^{(p+2)/p})$, it is enough to prove that $\max \varphi^{(p)}(X_n) \leqslant O(n^{p+2})$. We do it in the next lemma.

Lemma 15. *The maximum value of $\varphi^{(p)}(T)$ on $\mathcal{UT}_n$, $\mathcal{T}_n$ or $\mathcal{BT}_n$ is reached at the rooted caterpillars, and its value is in $\Theta(n^{p+2})$.*

Proof. Arguing as in the case $p = 1$, we have that the maximum value of $\varphi^{(p)}(T)$ on $\mathcal{UT}_n$ is reached on trees in $\mathcal{T}_n$, because if we replace each nested taxon in a tree by a new leaf labeled with the same taxon as in Fig. 13, the value of $\varphi^{(p)}$ increases. On the other hand, if a tree $T \in \mathcal{T}_n$ contains a node with $k \geqslant 3$ children, as in the left hand side of Fig. 14, and we replace its subtree rooted at this node as described in the right hand side of Fig. 14, we obtain a new tree $T' \in \mathcal{T}_n$ with larger $\varphi^{(p)}$ value: the values of $\varphi(i,j)^p$ for $i, j \in L(T_1) \cup \cdots \cup L(T_{k-1})$ increase, and the other values of $\varphi(i,j)^p$ do not change. This implies that for every non-binary phylogenetic tree $T \in \mathcal{T}_n$, there always exists a binary phylogenetic tree $T' \in \mathcal{BT}_n$ such that $\varphi^{(p)}(T') > \varphi^{(p)}(T)$ and in particular that the maximum value of $\varphi^{(p)}(T)$ on $\mathcal{UT}_n$ is actually reached on $\mathcal{BT}_n$.

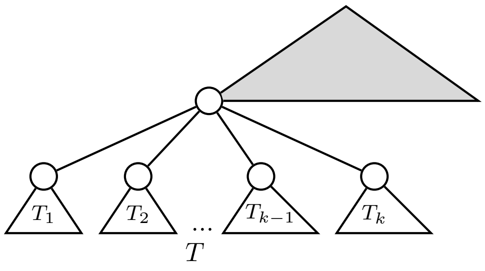

Figure 14: $\varphi^{(p)}(T') > \varphi^{(p)}(T)$.

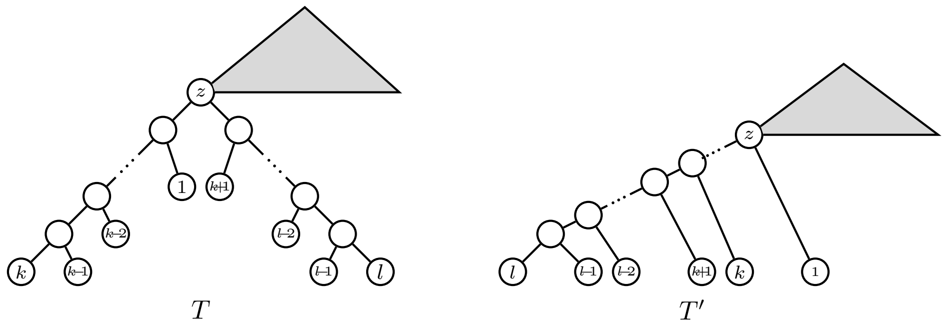

Figure 15: $\varphi^{(p)}(T') > \varphi^{(p)}(T)$.

Let now $T \in \mathcal{BT}_n$ and assume that it is not a caterpillar. Therefore, it has an internal node z of largest depth without any leaf child; in particular, all internal descendant nodes of z have some leaf child. Thus, and up to a relabeling of its leaves, T has the form represented in the left hand side of Fig. 15, for some $k \geqslant 2$ and some $l \geqslant k+2$. Consider then the tree T' depicted in right hand side of Fig. 15, where the grey triangle represents the same tree in both sides. It turns out that $\varphi^{(p)}(T') - \varphi^{(p)}(T) > 0$. Indeed, if q denotes the depth of the node z in both trees, then

$$\varphi_{T'}(i,j)^p - \varphi_T(i,j)^p = \begin{cases} (q+i)^p - (q+i+1)^p & \text{if } 1 \leqslant i = j \leqslant k-1 \\ 0 & \text{if } i = j = k \\ (q+i)^p - (q+i-k+1)^p & \text{if } k+1 \leqslant i = j \leqslant l-1 \\ (q+l-1)^p - (q+l-k)^p & \text{if } i = j = l \\ (q+i-1)^p - (q+i)^p & \text{if } 1 \leqslant i < j \leqslant k \\ (q+i-1)^p - (q+i-k)^p & \text{if } k+1 \leqslant i < j \leqslant l \\ (q+i-1)^p - q^p & \text{if } 1 \leqslant i \leqslant k < j \leqslant l \\ 0 & \text{otherwise} \end{cases}$$

Therefore,

$$\begin{aligned}
\varphi^{(p)}(T') - \varphi^{(p)}(T) &= \sum_{i=1}^{k-1}\big((q+i)^p - (q+i+1)^p\big) + \sum_{i=k+1}^{l-1}\big((q+i)^p - (q+i-k+1)^p\big) \\
&\quad +(q+l-1)^p - (q+l-k)^p + \sum_{i=1}^{k-1}(k-i)\big((q+i-1)^p - (q+i)^p\big) \\
&\quad + \sum_{i=k+1}^{l-1}(l-i)\big((q+i-1)^p - (q+i-k)^p\big) + \sum_{i=1}^{k}(l-k)\big((q+i-1)^p - q^p\big) \\
&= (q+1)^p - (q+k)^p + \sum_{i=1}^{l-k-1}\big((q+k+i)^p - (q+1+i)^p\big) \\
&\quad +(q+l-1)^p - (q+l-k)^p + \sum_{i=1}^{k-1}(k-i)\big((q+i-1)^p - (q+i)^p\big) \\
&\quad + \sum_{i=1}^{l-k-1}(l-k-i)\big((q+k+i-1)^p - (q+i)^p\big) + \sum_{i=1}^{k}(l-k)\big((q+i-1)^p - q^p\big)
\end{aligned}$$

To prove that this sum is non-negative, let us write it as

$$\varphi^{(p)}(T') - \varphi^{(p)}(T) = S_1 + S_2 + S_3,$$

where

$$\begin{aligned}
S_1 &= \sum_{i=1}^{k-1}(k-i)\big((q+i-1)^p - (q+i)^p\big) + \sum_{i=1}^{k}(l-k)\big((q+i-1)^p - q^p\big) \\
S_2 &= \sum_{i=1}^{l-k-1}\big((q+k+i)^p - (q+1+i)^p\big) + \sum_{i=1}^{l-k-1}(l-k-i)\big((q+k+i-1)^p - (q+i)^p\big) \\
S_3 &= (q+1)^p - (q+k)^p + (q+l-1)^p - (q+l-k)^p
\end{aligned}$$

Then

$$
\begin{aligned}
S_1 &= \sum_{i=1}^{k-1}(k-i)\big((q+i-1)^p-(q+i)^p\big)+\sum_{i=1}^{k}(l-k)\big((q+i-1)^p-q^p\big),\\
&= \sum_{i=1}^{k-1}(k-i)(q+i-1)^p-\sum_{i=1}^{k-1}(k-i)(q+i)^p+\sum_{i=1}^{k}(l-k)\big((q+i-1)^p-q^p\big),\\
&= \sum_{i=1}^{k-1}(k-i)(q+i-1)^p-\sum_{i=2}^{k}(k-i+1)(q+i-1)^p+(l-k)\sum_{i=1}^{k}(q+i-1)^p-k(l-k)q^p,\\
&= \sum_{i=1}^{k-1}(l-k-1)(q+i-1)^p+kq^p-(q+k-1)^p+(l-k)(q+k-1)^p-k(l-k)q^p,\\
&= (l-k-1)\sum_{i=1}^{k}\big((q+i-1)^p-q^p\big)>0\\
S_2 &= \sum_{i=1}^{l-k-1}\big((q+k+i)^p-(q+1+i)^p\big)+\sum_{i=1}^{l-k-1}(l-k-i)\big((q+k+i-1)^p-(q+i)^p\big)\\
&= \sum_{i=1}^{l-k-1}\big((q+k+i)^p-(q+1+i)^p\big)+\sum_{i=0}^{l-k-1}(l-k-i-1)\big((q+k+i)^p-(q+i+1)^p\big)\\
&= \sum_{i=1}^{l-k-1}(l-k-i)\big((q+k+i)^p-(q+1+i)^p\big)+(l-k-1)\big((q+k)^p-(q+1)^p\big)\\
&> (l-k-1)\big((q+k)^p-(q+1)^p\big).
\end{aligned}
$$

and therefore

$$
\begin{aligned}
\varphi^{(p)}(T')-\varphi^{(p)}(T) &= S_1+S_2+S_3\\
&> (l-k-1)\big((q+k)^p-(q+1)^p\big)+(q+1)^p-(q+k)^p+(q+l-1)^p-(q+l-k)^p\\
&= (l-k-2)\big((q+k)^p-(q+1)^p\big)+(q+l-1)^p-(q+l-k)^p>0.
\end{aligned}
$$

This implies that no tree other than a rooted caterpillar can have the largest $\varphi^{(p)}$ value in $\mathcal{BT}_n$, and hence also in $\mathcal{T}_n$ and $\mathcal{UT}_n$.

Finally, if K_n denotes the rooted caterpillar with n leaves in Fig. 12.(c),

$$
\varphi_{K_n}(i,j)^p=\begin{cases}(n-1)^p & \text{if } i=j=1\\ (n-i+1)^p & \text{if } 2\leqslant i=j\leqslant n\\ (n-j)^p & \text{if } 1\leqslant i<j\leqslant n\end{cases}
$$

and thus

$$
\begin{aligned}
\varphi^{(p)}(K_n) &= (n-2)\cdot 1^p+(n-3)\cdot 2^p+\cdots+2\cdot(n-3)^p+1\cdot(n-2)^p\\
&\qquad +1^p+2^p+\cdots+(n-2)^p+(n-1)^p+(n-1)^p\\
&= (n-1)\cdot 1^p+(n-2)\cdot 2^p+\cdots+3\cdot(n-3)^p+2\cdot(n-2)^p+(n-1)^p+(n-1)^p\\
&= \sum_{k=1}^{n-1}(n-k)\cdot k^p+(n-1)^p
\end{aligned}
$$

Now, it turns out that

$$
\sum_{k=1}^{n-1}k^m=\frac{1}{m+1}n^{m+1}+O(n^m). \tag{1}
$$

This property is well known for natural numbers $m \in \mathbb{N}$ [5]. For arbitrary real numbers $m > 0$, it derives from the fact that

$$\int_1^{n-1} (x-1)^m dx \leqslant \sum_{k=1}^{n-1} k^m \leqslant \int_1^{n-1} x^m dx,$$

and then

$$\int_1^{n-1} (x-1)^m dx = \frac{1}{m+1}(n-2)^{m+1} = \frac{1}{m+1} n^{m+1} + O(n^m)$$
$$\int_1^{n-1} x^m dx = \frac{1}{m+1}(n-1)^{m+1} = \frac{1}{m+1} n^{m+1} + O(n^m)$$

So, by identity (1), we have that

$$\sum_{k=1}^{n-1}(n-k)\cdot k^p + (n-1)^p = n\sum_{k=1}^{n-1} k^p - \sum_{k=1}^{n-1} k^{p+1} + O(n^p) = \Big(\frac{1}{p+1} - \frac{1}{p+2}\Big) n^{p+2} + O(n^{p+1})$$

and hence $\varphi^{(p)}(K_n)$ is in $\Theta(n^{p+2})$. □

Therefore, $O(n^{(p+2)/p}) \leqslant \Delta_p(X_n) \leqslant O(n^{(p+2)/p})$, which shows that the diameter of $d_{\varphi,p}$ on $\mathcal{UT}_n$, $\mathcal{T}_n$ and $\mathcal{BT}_n$ is indeed in $\Theta(n^{(p+2)/p})$.

We finally prove the case $p = 0$, which needs a completely different argument.

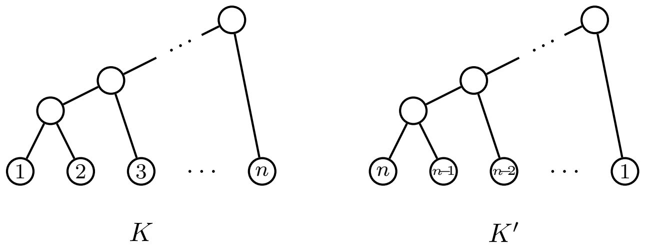

Figure 16: The caterpillars used in the proof of Lemma 16.

Lemma 16. *The diameter of $d_{\varphi,0}$ on $\mathcal{UT}_n$, $\mathcal{T}_n$ and $\mathcal{BT}_n$ is in $\Theta(n^2)$.*

Proof. Since the cophenetic vector of a tree $T \in \mathcal{UT}_n$ lies in $\mathbb{R}^{n(n+1)/2}$, it is clear that $d_{\varphi,0}(T_1,T_2) \leqslant n(n+1)/2$, for every $T_1, T_2 \in \mathcal{UT}_n$. Now, consider the pair of rooted caterpillars with n leaves depicted in Fig. 16. We have that

$$\begin{array}{lll}
\varphi_K(i,j) = n-j & \varphi_{K'}(i,j) = i-1 & \text{for every } 1 \leqslant i < j \leqslant n \\
\varphi_K(i,i) = n-i+1 & \varphi_{K'}(i,i) = i & \text{for every } 2 \leqslant i \leqslant n-1 \\
\varphi_K(1,1) = n-1 & \varphi_{K'}(1,1) = 1 & \\
\varphi_K(n,n) = 1 & \varphi_{K'}(n,n) = n-1 &
\end{array}$$

This shows that the number of pairs (i,j), $1 \leqslant i \leqslant j \leqslant n$, such that $\varphi_K(i,j) = \varphi_{K'}(i,j)$ is at most $(n+1)/2$, and therefore that $d_{\varphi,0}(K,K')$ is at least $(n^2-1)/2$. So, the diameter of $d_{\varphi,0}$ on $\mathcal{UT}_n$ is bounded from above

by $O(n^2)$, and its diameter on $\mathcal{BT}_n$ is bounded from below by $O(n^2)$, which implies that the diameter of $d_{\varphi,0}$ on $\mathcal{UT}_n$, $\mathcal{T}_n$ and $\mathcal{BT}_n$ is in $\Theta(n^2)$. $\square$

References

1. A. Mir, F. Rosselló, L. Rotger, A new balance index for phylogenetic trees. arXiv:1202.1223v1 [q-bio.PE] (2012), submitted to Mathematical Biosciences.

2. R. Morris, Some theorems on sorting. SIAM Journal of Applied Mathematics 17 (1969), 1–6.

3. M. J. Sackin, "Good" and "bad" phenograms. Systematic Zoology, 21 (1972), 225–226.

4. K.T. Shao, R. Sokal, Tree balance. Systematic Zoology, 39 (1990), 226–276.

5. E. W. Weisstein, Power Sum. From MathWorld–A Wolfram Web Resource. http://mathworld.wolfram.com/PowerSum.html
